# Enhanced Nitric Oxide Delivery Through Self‐Assembling Nanoparticles for Eradicating Gram‐Negative Bacteria

**DOI:** 10.1002/adhm.202403046

**Published:** 2024-09-12

**Authors:** Xiangfeng Lai, Lei Yu, Xiangyi Huang, Wil Gardner, Sarah E. Bamford, Paul J. Pigram, Shuhong Wang, Anton P. Le Brun, Benjamin W. Muir, Jiangning Song, Yajun Wang, Hsien‐Yi Hsu, Philip Wai Hong Chan, Hsin‐Hui Shen

**Affiliations:** ^1^ Department of Materials Science and Engineering Faculty of Engineering Monash University Clayton Victoria 3800 Australia; ^2^ School of Chemistry Monash University Clayton VIC 3800 Australia; ^3^ Centre for Materials and Surface Science and Department of Mathematical and Physical Sciences La Trobe University Bundoora 3086 Australia; ^4^ Australian Centre for Neutron Scattering Australian Nuclear Science and Technology Organisation Lucas Heights NSW 2232 Australia; ^5^ CSIRO Manufacturing Clayton VIC 3168 Australia; ^6^ Infection and Immunity Program Monash Biomedicine Discovery Institute and Department of Microbiology Monash University Clayton VIC 3800 Australia; ^7^ College of Chemistry & Materials Engineering Wenzhou University Shanghai Wenzhou 325027 China; ^8^ School of Energy and Environment & Department of Materials Science and Engineering City University of Hong Kong Kowloon Tong Hong Kong; ^9^ Shenzhen Research Institute of City University of Hong Kong Shenzhen 518057 China

**Keywords:** antimicrobial resistance, bacterial membrane, neutron, nitric oxide, self‐assembly

## Abstract

In the current battle against antibiotic resistance, the resilience of Gram‐negative bacteria against traditional antibiotics is due not only to their protective outer membranes but also to mechanisms like efflux pumps and enzymatic degradation of drugs, underscores the urgent need for innovative antimicrobial tactics. Herein, this study presents an innovative method involving the synthesis of three furoxan derivatives engineered to self‐assemble into nitric oxide (NO) donor nanoparticles (FuNPs). These FuNPs, notably supplied together with polymyxin B (PMB), achieve markedly enhanced bactericidal efficacy against a wide spectrum of bacterial phenotypes at considerably lower NO concentrations (0.1–2.8 µg mL^−1^), which is at least ten times lower than the reported data for NO donors (≥200 µg mL^−1^). The bactericidal mechanism is elucidated using confocal, scanning, and transmission electron microscopy techniques. Neutron reflectometry confirms that FuNPs initiate membrane disruption by specifically engaging with the polysaccharides on bacterial surfaces, causing structural perturbations. Subsequently, PMB binds to lipid A on the outer membrane, enhancing permeability and resulting in a synergistic bactericidal action with FuNPs. This pioneering strategy underscores the utility of self‐assembly in NO delivery as a groundbreaking paradigm to circumvent traditional antibiotic resistance barriers, marking a significant leap forward in the development of next‐generation antimicrobial agents.

## Graphical abstract

This study presents an innovative approach to combat antibiotic‐resistant Gram‐negative bacteria by utilizing self‐assembling nanoparticles that deliver nitric oxide (NO). These nanoparticles, combined with polymyxin B, significantly enhance antibacterial efficacy at lower NO concentrations, disrupting bacterial membranes and reducing resistance development. The findings offer a promising pathway for next‐generation antimicrobial therapies.

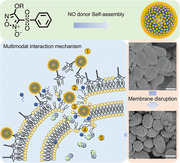

## 1 Introduction

The global health crisis exacerbated by antimicrobial resistance (AMR) is a pressing concern, with Gram‐negative bacteria such as *Acinetobacter baumannii*, *Pseudomonas aeruginosa*, *Klebsiella pneumoniae* and *Escherichia coli* at the forefront of this challenge.^[^ ^1^ ^]^ These pathogens are not only pervasive across various healthcare settings but also significant contributors to the high morbidity and mortality rates associated with resistant infections.^[^ ^2^ ^]^ According to the World Health Organization, AMR was directly responsible for an estimated 1.27 million deaths globally in 2019, surpassing fatalities from diseases such as HIV or malaria. This positions AMR as a leading cause of death worldwide,^[^ ^3^ ^]^ emphasizing the critical necessity for innovative therapeutic approaches. Additionally, the significant increase in resistance to last‐line antibiotics like polymyxins among these pathogens underscores the critical challenge of developing effective treatments against such resilient bacteria.^[^ ^2^ ^]^

The exploration of gaseous nitric oxide (NO) as a countermeasure against bacterial infections, particularly those highly resistant to conventional antibiotics, signifies a transformative approach in antimicrobial methodologies.^[^ ^4^ ^]^ NO's ability to disrupt biofilms and exert direct bactericidal effects positions it as an effective broad‐spectrum agent, inducing oxidative and nitrosative stress within bacterial cells.^[^ ^4^, ^5^ ^]^ However, the volatile nature of NO presents challenges in dosage accuracy, storage, and delivery.^[^ ^6^ ^]^ To address these issues, recent efforts have focused on developing advanced NO delivery systems for controlled NO release by encapsulating or conjugating NO in nanocarriers.^[^ ^7^ ^]^ Despite these promising developments, several challenges remain. 1) The poor stability of most NO donors results in a rapid release of NO before reaching bacterial targets, which limits effectiveness and practical application.^[^ ^6b^ ^]^ 2) Current effective dosages of NO‐releasing nanoparticles that release NO at concentrations above 20 µg mL^−1^ have been found to induce toxicity, thus posing risks to mammalian cells.^[^ ^8^ ^]^ 3) There is a risk of bacteria developing resistance to NO, reflecting the broader issue of antibiotic resistance.^[^ ^9^ ^]^ 4) Precise delivery of NO to bacterial targets remains a significant challenge. These complexities underscore the importance of continued research into sophisticated NO delivery systems, crucial for unlocking its full antimicrobial potential.^[^ ^10^ ^]^

Our study pioneers a novel therapeutic approach to address the four pressing challenges by developing furoxan‐based compounds capable of self‐assembling into stable NO donor nanoparticles (FuNPs). Unlike traditional methods that involve active substance loading,^[^ ^11^ ^]^ our approach leverages the self‐assembly capabilities of FuNPs, which have exhibited notable stability across 72 h, maintaining a consistent NO release throughout this duration. These self‐assembled FuNPs not only boosts the delivery and effectiveness of NO but does so at low NO concentrations (0.1–2.8 µg mL^−1^). Furthermore, FuNPs supplying together with polymyxin B (PMB) (hereinafter referred to as PMB/FuNPs) presents a viable strategy to circumvent the development of antimicrobial resistance and demonstrates an enhancement of antibacterial activity, as evidenced by comprehensive in vitro assays. Neutron reflectometry has been instrumental to reveal that FuNPs specifically target a spectrum of Gram‐negative bacteria by specifically binding to polysaccharides on their outer membrane, compromising bacterial integrity and inducing a breach in their defense.

## 2 Results and Discussion

### 2.1 Synthesis and Characterization of Furoxan Derivatives

**Figure 1:**
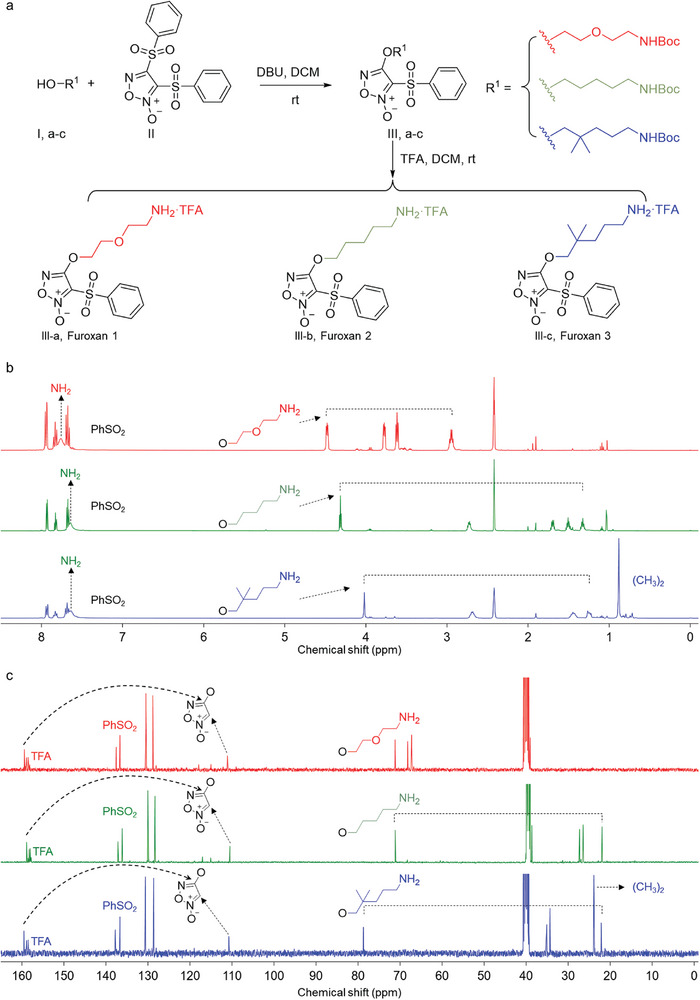
Synthesis and characterization of furoxan derivatives. a) Schematics of the synthetic route of furoxan derivatives. DBU, 1,8‐diazabicyclo(5.4.0) undec‐7‐ene; DCM, dichloromethane; rt, room temperature. TFA, trifluoroacetic acid. b) ^1^H‐NMR spectra of furoxan derivatives in DMSO‐d_6_ at 25 °C. c) ^13^C‐NMR spectra of furoxan derivatives in DMSO‐d_6_ at 25 °C.

To investigate the synthesis of furoxan derivatives with potential antibacterial properties, we meticulously chose three amino building blocks with comparable chain lengths to minimize the effects of chain length variability on antibacterial efficiency. These building blocks were 2‐(2‐aminoethoxy) ethanol (**I‐a**), 5‐amino‐2,2‐dimethylpentanol (**I‐b**), and 5‐amino‐1‐pentanol (**I‐c**), each selected for their synthetic feasibility and unique amino moieties (**Figure** **1**a). This deliberate selection aimed to ensure that any observed variations in antibacterial efficiency could be ascribed to the intrinsic properties of the compounds, rather than to differences in chain length. The synthesis was conducted through a simple, two‐step reaction at ambient temperature, proceeding overnight to yield ≈52% for the derivatives **III‐a‐c**: 4‐(2‐(2‐aminoethoxy)ethoxy)(3‐phenylsulfonyl)furoxan (Furoxan‐1), 4‐((5‐amino‐2,2‐dimethylpentyl)oxy)(3‐phenylsulfonyl)furoxan (Furoxan‐2), and 4‐((5‐aminopentyl)oxy)(3‐phenylsulfonyl)furoxan (Furoxan‐3). The methodology's high efficiency and yield underscore its practicality and effectiveness. Subsequent characterization of the synthesized derivatives was performed using ^1^H and ^13^C NMR spectroscopy (Figure 1b,c). The ^1^H NMR spectra revealed significant peaks at ≈7.8 ppm, indicative of aromatic protons, whose downfield shift suggested the electron‐withdrawing nature of the phenyl group and hinted at the presence of a monosubstituted benzene ring (PhSO_2_–). Evidence of aliphatic protons was observed with peaks below 3 ppm, attributable to the methyl and methylene groups. Peaks in the 3–5 ppm range suggested protons adjacent to heteroatoms (O or N), indicative of a deshielding effect and a downfield shift. Notably, for Furoxan‐3, a sharp peak ≈0.95 ppm was indicative of the methyl group protons. Integration of these spectra (Figures S1–S3, Supporting Information) supported the proposed structures of the furoxan derivatives, with the relative peak areas correlating with the expected number of protons in each chemical environment. Additionally, the corresponding ^13^C NMR spectra (Figure 1c) further confirmed the successful synthesis of the furoxan derivatives and provides the foundations for future investigations into their antibacterial applications.

### 2.2 Formulation and Characterization of FuNPs

**Figure 2:**
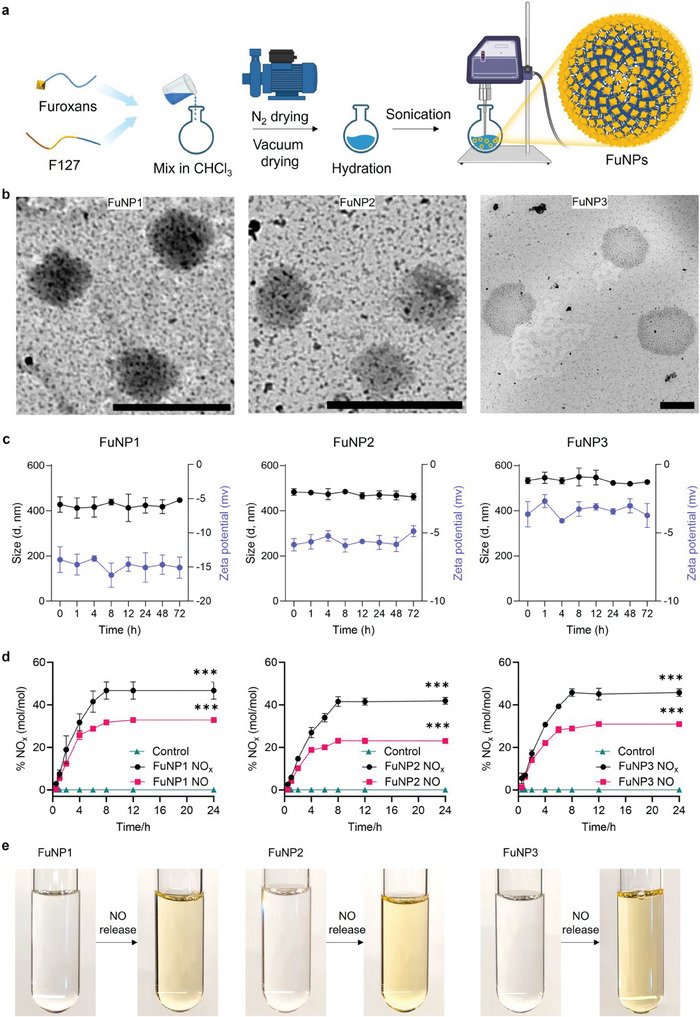
Formulation and characterization of FuNPs. a) Schematic representation of self‐assembling route of furoxan derivatives to form FuNPs. b) TEM images showing the spherical morphology (scale bar: 500 nm). c) Stability test of FuNPs in 1X phosphate buffer solution (PBS buffer, pH 7.4, 37 °C) via dynamic light scattering across 72 h. d) NO_x_ release kinetics (black circles) measured in the presence of L‐cysteine and NO release kinetics (red circles) measured using the DAN‐based assay of FuNPs over time at 37 °C (1X PBS, pH 7.4). The results are expressed as the percent (% mol/mol) of NO or NO_x_ released with respect to the quantity of parent furoxan compound. Symbols represent data from the three replicates, and error bars represent the standard deviation from the mean. e) Representative appearance of FuNP solutions before (clear transparent) and after NO releasing (yellow). Statistical significance was determined with a Student's t‐test compared to the controls without adding L‐cysteine. ***p < 0.001.

Intriguingly, upon mixing with Pluronic F127, furoxan derivatives spontaneously self‐assembled into furoxan nanoparticles (FuNPs) through a sequential methodology that incorporated drying, hydration, and sonication processes (**Figure** **2**a). The self‐assembly of furoxans is primarily driven by the synergistic effect of hydrophobic interactions and hydrogen bonding, as dictated by their molecular structure. Each furoxan monomer is designed with a hydrophobic aromatic group, which promotes aggregation in aqueous environments by minimizing unfavorable interactions with water. Additionally, the monomers feature amide groups capable of forming hydrogen bonds. These hydrogen bonding interactions facilitate the cohesive assembly of the monomers into stable, ordered supramolecular structures. The FuNPs assembly was subsequently confirmed via transmission electron microscopy (TEM), where spherical morphologies were observed (Figure 2b). Further analysis concerning the stability of the nanoparticles revealed a narrow size distribution range on the order of 400–550 nm (Figure 2c; Tables s1–s3, Supporting Information), affirming the TEM results and indicating a homogeneous size distribution crucial for their application in biological contexts. Additionally, stability assessments demonstrated that the FuNPs maintained their structural integrity across a 72‐h period. Surface charge evaluations further revealed that all FuNPs possessed a negative zeta potential, underscoring their potential stability in biological systems (Figure 2c; Tables S1–S3, Supporting Information). To investigate biocompatibility, human HeLa cells were incubated with FuNPs at a concentration of 128 µg mL^−1^, and cell viability was assessed at various time points to evaluate the safety of NO release kinetics (Figure S4, Supporting Information). A subsequent MTT assay revealed all three FuNPs are non‐cytotoxic within 72 h. These results demonstrate that the formulations are biocompatible and safe for use in medical applications.

Interestingly, the initial colorless FuNP solution underwent a notable color change to yellow following the release of NO (Figure 2e). This color transformation, coupled with the undetectable size distribution of FuNPs, suggests that the structure of FuNPs was significantly altered post‐NO release. To elucidate the kinetics of NO release by FuNPs, we examined NO liberation in the presence of thiols, such as L‐cysteine, using nitrite production as an indicator of NO dynamics in phosphate buffer solution (PBS) under physiologically relevant conditions (pH 7.4, 37 °C).^[^ ^12^ ^]^ Since thiols are commonly produced by bacteria in the millimolar range, and this concentration range is generally sufficient to mediate the reduction and subsequent opening of the furoxan ring in FuNPs, facilitating NO release, they are highly relevant in the context of bacterial infection treatments.**^[^** ^13^ ^]^ This mimics physiological conditions where endogenous thiols like L‐cysteine trigger NO release, allowing us to accurately measure the controlled release kinetics of NO from the nanoparticles. The quantification of released NO was accomplished through the Griess assay and a 2,3‐diaminonaphthalene (DAN)‐based chemical assay, which hinges on the instantaneous reaction of NO with oxygen to form dinitrogen trioxide, which subsequently reacts with DAN to yield highly fluorescent 2,3‐naphthotriazole (NAT), measurable by its fluorescence intensity.^[^ ^14^ ^]^ Our investigations into the release kinetics of NO_x_ from FuNPs under physiologically relevant conditions revealed spontaneous liberation of both NO and nitrite (NO_2_^−^), unveiling distinct NO_x_ production profiles (Figure 2d; Figure S5, Supporting Information). Specifically, FuNP1 and FuNP3 resulted in a rapid initial NO release of 28% of the total NO_x_ within the first 6 h, which suggests that they rapidly decomposed upon exposure to physiological conditions, resulting in a swift initial burst of NO release which quickly plateaus after 8 h as these nanoparticles exhausted their NO content, constituting 32% of the total detected NO_x_ (45%) within 24 h. Conversely, FuNP2 shows a slower and more gradual initial release of NO and NO_2_^−^, with an almost linear liberation pattern over the first 6 h, culminating in 23% of the detected NO_x_ species as NO and 19% as NO_2_^−^ by the 24‐h mark (Figure 2d). These release kinetics of NO_x_ from FuNPs suggest that the final release of NO_x_ is ≈45% of the total potential NO_x_ content, likely due to incomplete transformation of the furoxan within the nanoparticles.

### 2.3 Antibacterial Activities of FuNPs in vitro

#### 2.3.1 PMB/FuNPs Synergistically Kill Bacteria

The antibacterial efficacy of FuNPs was initially assessed using the minimum inhibitory concentration (MIC) assay against a panel of 23 PMB‐susceptible and ‐resistant Gram‐negative bacteria, which included strains of *A. baumannii*, *P. aeruginosa*, *K. pneumoniae*, and *E. coli*, employing the double broth dilution method (Table S4, Supporting Information). Although the furoxan derivatives showed potential as antimicrobials, it was notable that effective bacterial inhibition by FuNPs required high dosages (≥256 µg mL^−1^), with a particularly enhanced effect observed against *E. coli* strains. Subsequent analysis utilizing the checkerboard assay demonstrated that PMB at 1/4 × MIC supplied together with FuNPs significantly reduced the fractional inhibitory concentrations (FICs) across all 23 tested strains (Tables S5–S7, Supporting Information), indicating substantial growth inhibition. Notably, all PMB/FuNPs exhibited synergistic effects against the tested Gram‐negative bacteria, with only 1/4 × MIC of PMB and 4–128 µg mL^−1^ FuNPs (NO dose ≈ 0.1–2.8 µg mL^−1^ after highest 32% release), which was significantly lower than the previously reported data for NO‐releasing nanoparticles (≥200 µg mL^−1^), either standalone or supplied together with conventional antibiotics.^[^ ^4^, ^15^ ^]^ In contrast, the bulk form of furoxans required twofold to eightfold higher concentrations to achieve similar effects (Table S8, Supporting Information), highlighting the critical role that nanoformulation plays in enhancing the interaction with and disruption of bacterial membranes. The improved performance of the nanoformulation is likely due to its ability to more effectively interact with bacterial membranes, leading to more efficient NO release and greater antimicrobial efficacy. To evaluate the potential advantages of FuNPs over other current antibiotics when supplied together with PMB (PMB/antibiotic), three different antibiotics that target Gram‐negative bacteria, including Ampicillin, Aztreonam and Doripenem, were selected for testing against the bacteria (Tables S9–S11, Supporting Information). The results showed that PMB/antibiotic did not enhance antibacterial activity against all tested bacterial strains. This is consistent with our previous study, where we demonstrated that only membrane‐targeting antimicrobials supplied together with PMB significantly enhanced antibacterial killing efficacy.^[^ ^16^ ^]^ This enhanced efficacy of PMB/FuNPs could be attributed to the self‐assembly characteristics and the membrane targeting properties of FuNPs, which not only circumvent the NO instability in biological media but also significantly boost NO effectiveness. Surprisingly, after inducing NO release from all FuNPs, PMB supplied together with NO‐released FuNP exhibited indifferent efficacy to the same bacteria as tested by PMB/FuNPs (Table S12, Supporting Information), underscoring the necessity of the self‐assembly of the NO‐releasing nanoparticles and highlights the broad‐spectrum efficacy of the PMB/FuNP antimicrobial approach.

**Figure 3:**
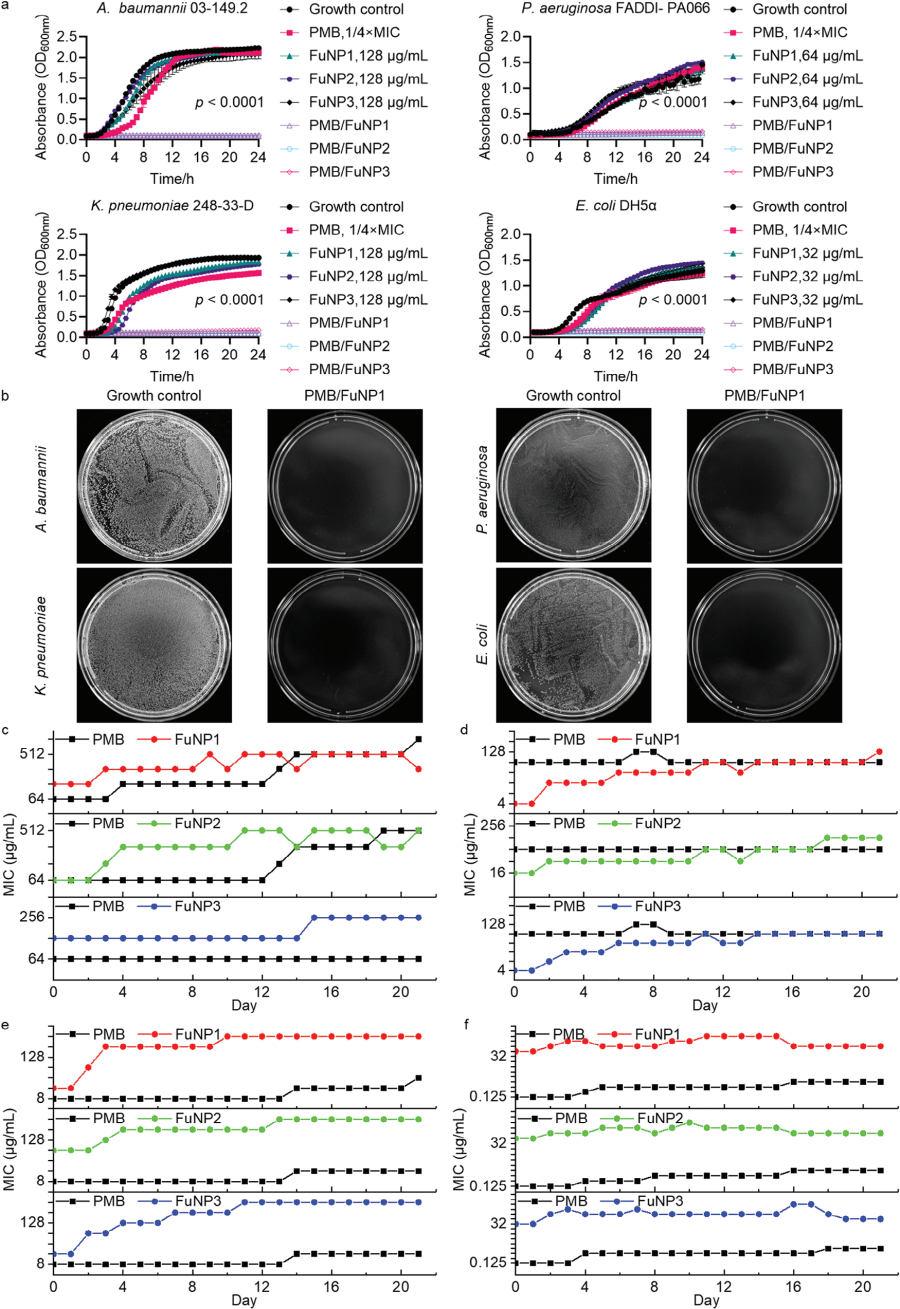
In vitro antibacterial activity of FuNPs. a) Growth curves (*n = 3*) for cultures of *A. baumannii*, *P. aeruginosa*, *K. pneumoniae*, and *E. coli* in the presence of polymyxin B (PMB, 1/4 × MIC, Table S1, Supporting Information), FuNPs and PMB/FuNPs. b) Representative images of *A. baumannii*, *P. aeruginosa*, *K. pneumoniae*, and *E. coli* colonies cultured on the agar plates after 24 h of PMB/FuNP1 treatment in a). Resistance acquisition (n = 2) of c) *A. baumannii*, d) *P. aeruginosa*, e) *K. pneumoniae* and f) *E. coli* during serial passage for 21 days in the presence of 1/4 × MIC levels of PMB in cation adjusted Mueller–Hinton broth. Statistically significant differences were analyzed by student t‐test between the control group and the PMB/FuNP treatments.

Subsequent exploration of growth curves for these bacterial isolates post‐treatment showed that, although cultures treated with PMB at 1/4 × MIC and FuNPs at concentrations of 32–128 µg mL^−1^ exhibited slower growth compared to untreated controls, they reached saturation after 24 h (**Figure** **3**a). In contrast, cultures subjected to the combined treatment of PMB and FuNPs demonstrated significantly enhanced bacterial killing activity, as evidenced by the rescued growth of *A. baumannii*, *P. aeruginosa*, *K. pneumoniae*, and *E. coli* within the same timeframe. This observation was corroborated by the spread plate method (Figure 3b; Figure S6, Supporting Information), which aligned with the previously mentioned findings, highlighting the limited antibacterial capacity of PMB and FuNP monotherapies while underscoring the significant efficacy of the PMB/FuNPs therapy in combating bacterial growth.

#### 2.3.2 PMB/FuNPs Prevent Development of Resistance

Next, we explored whether the concurrent administration of PMB at 1/4 × MIC supplied together with FuNPs could impede the evolution of antibiotic resistance in laboratory settings (Figure 3c‐f). To this end, we conducted experiments on *A. baumannii*, *P. aeruginosa*, *K. pneumoniae*, and *E. coli* using three distinct combinations: PMB/FuNP1, PMB/FuNP2, and PMB/FuNP3. These bacterial strains were cultivated in the presence of both the combination therapies and their individual components. Remarkably, after 21 days of serial passaging, bacterial populations subjected to monotherapy exhibited substantial increases in MIC levels for PMB and FuNPs compared to their ancestral strains (Tables S13 and S14, Supporting Information). In stark contrast, the co‐administration of PMB and FuNPs markedly decelerated the development of resistance (Figure 3c‐f). This observation suggests that the combined treatment imposes a higher fitness cost on resistance mutations, potentially explaining its efficacy.

### 2.4 Antimicrobial Mechanism

#### 2.4.1 PMB/FuNPs Disrupt Bacterial Membrane

Building on the observed antibacterial efficacy of PMB/ FuNPs against Gram‐negative bacteria (Figure 3), we selected each bacterium for an in‐depth investigation of the antibacterial mechanisms of PMB/FuNPs in vitro. This exploration was structured around three therapeutic groups: PMB monotherapy, FuNP monotherapy, and combined PMB/FuNPs treatment. Given the comparable effectiveness of the three types of FuNPs, we chose FuNP1 for our mechanistic studies. Initially, dead/live staining visualized through confocal laser scanning microscopy underscored the anticipated antibacterial effect (**Figure** **4**a; Figure S7, Supporting Information). This staining technique distinguishes bacteria with intact cell membranes, which appear green due to SYTO‐9 staining (λ_Ex/Em_ = 483/503 nm), from those with compromised membranes, which turn red from propidium iodide staining (λ_Ex/Em_ = 535/617 nm). Confocal imagery revealed that, under PMB/FuNP1 treatment, all bacteria (*A. baumannii*, *P. aeruginosa*, *K. pneumoniae*, and *E. coli*) showed red fluorescence (growth control, Figure 4a), indicating cell death, while cells in other treatment groups predominantly exhibited green fluorescence (Figure S7a, Supporting Information), highlighting the superior destructiveness of PMB/FuNP1 against the Gram‐negative bacteria.

Figure 4Visualization of the changes of bacteria after incubation with PMB/FuNP1 in vitro. a) Representative confocal fluorescence images for a live/dead assay of 4 Gram‐negative bacteria after incubation with PMB/FuNP1 at 37 °C for 4 h. Scale bar: 5 µm. b) SEM images of 4 Gram‐negative bacteria after PMB/FuNP1 treatment at 37 °C for 4 h. The rough surface of the bacteria represents the typical disruption of bacterial morphology compared to the control. Scale bar: 500 nm. c) TEM images of 4 Gram‐negative bacteria after PMB/FuNP1 treatment at 37 °C for 4 h. The disruption of the membrane and the leakage of the intracellular contents represent the typical antibacterial activity of this treatment compared to the control. Scale bar: 500 nm. d) Intensities of CHO^+^, CH_3_O^+^, C_2_H_2_O^+^, C_2_H_3_O^+^, CH_3_O_2_^+^ and C_3_H_5_O2^+^ ToF‐SIMS peaks, for the untreated (control), FuNP1, PMB and PMB/FuNP1 treated bacteria. Intensities were normalized to primary ion dose for each measurement. Line plots show the mean intensity across all replicates, with shaded error bars showing standard error (*n = 15* for control, FuNP1, PMB, and *n = 14* for PMB/FuNP1). Inset bar plots showing mean summed peak intensities across replicates, with error bars again showing standard error.

**Figure:**
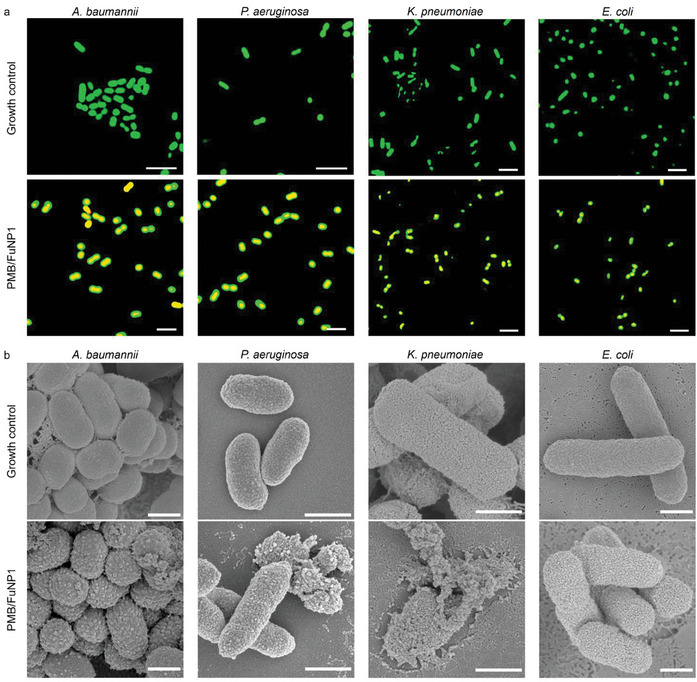

**Figure:**
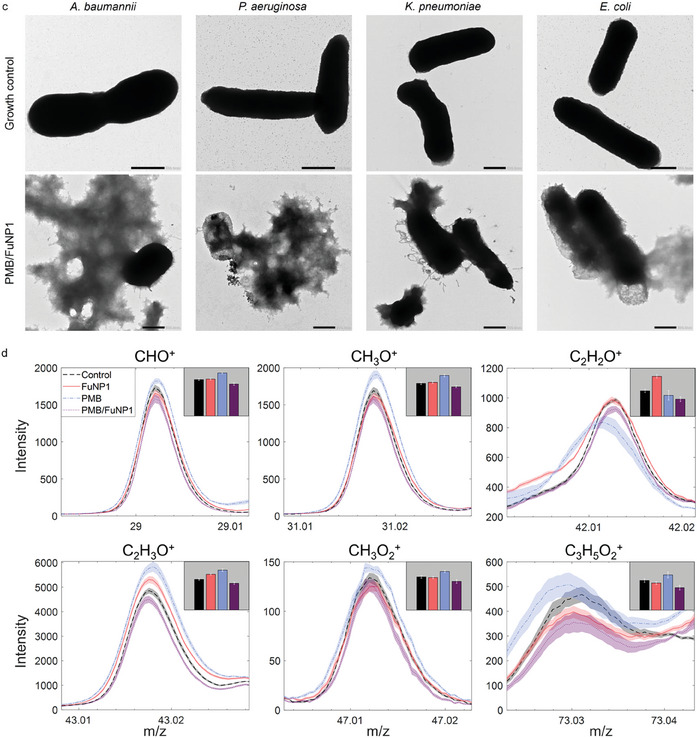

#### 2.4.2 PMB/FuNPs Induce Membrane Damage

Further supporting evidence was obtained from SEM images, which illustrated the morphological alterations in bacterial cells before and after treatment (Figure 4b; Figure S7b, Supporting Information). Untreated bacteria showcased a smooth and intact surface, whereas those exposed to PMB/FuNP1 treatment underwent significant morphological changes; the bacterial cell membranes were noticeably damaged and distorted, with the surfaces turning rough and cell lysis evident. In contrast, bacteria treated with either PMB or FuNP1 alone maintained their intact morphology (Figure S7b, Supporting Information). To investigate the leakage of intracellular components, we prepared samples for TEM imaging. Untreated cells displayed clear and intact membrane boundaries (Figure 4c). However, cells treated with PMB/FuNP1 exhibited pronounced rupture of the bacterial cell membrane, indicative of strong bactericidal activity, whereas cells treated with PMB or FuNP1 alone showed intact membranes, further demonstrating the potent antibacterial action of the PMB/FuNP1 combination (Figure S7c, Supporting Information).

#### 2.4.3 PMB/FuNPs Target Polysaccharides

Following the observation of morphological alterations in bacterial surface as a result of the treatment through microscopic studies, time‐of‐flight secondary ion mass spectrometry (ToF‐SIMS) was used to explore the interaction between FuNP1, PMB and PMB/FuNP1 with the bacterial cell surface in vitro. We acquired spectra from bacteria treated with FuNP1, PMB and PMB/FuNP1, as well as untreated bacteria (control, Figure 4d black curve). We focus on the CHO^+^, CH_3_O^+^, C_2_H_2_O^+^, C_2_H_3_O^+^, CH_3_O_2_^+^ and C_3_H_5_O_2_^+^ changes, which are indicative of polysaccharides presence on bacterial surfaces (Figure 4d). The bar plots in the corner depict the mean summed peak intensity across the CHO^+^, CH_3_O^+^, C_2_H_2_O^+^, C_2_H_3_O^+^, CH_3_O_2_^+^ and C_3_H_5_O_2_^+^ curves, providing a comparative measure of their abundance. Treatment with FuNP1 alone showed a relatively minor impact on the peak intensities, except for notable increases in the C_2_H_2_O^+^ and C_2_H_3_O^+^ (Figure 4d, red). Conversely, exposure to PMB alone resulted in a general elevation of these ion intensities, excluding C_2_H_2_O^+^, likely due to the antibiotic's composition rich in C, H and O (Figure 4d, blue). In contrast, the PMB/FuNP1 treatment led to a widespread reduction in the intensity of all measured ion fragments (Figure 4d, purple). The observation possibly suggests that the PMB/FuNP1 treatment may have reduced the accessible surface area of the bacterial membrane, predominantly composed of polysaccharides, to the primary ion beam, either by damage to the bacterial membranes, colocalization/attachment of the PMB/FuNP1 with the bacterial surface or some other mechanism.

#### 2.4.4 PMB/FuNPs Trigger Oxidative Stress

The effects of FuNPs and their released NO on membrane permeability were further assessed using 2′,7′‐dichlorodihydrofluorescein diacetate for sensitive detection of oxidative stress within bacterial cells (Figure S8, Supporting Information). By measuring the fluorescence intensity, the relationship between NO‐induced oxidative stress and the efficacy of different treatments was evaluated. At 1/4 × MIC, PMB partially perturbs the outer membrane of Gram‐negative bacteria, as evidenced by increased fluorescence due to elevated oxidative stress, indicating enhanced membrane permeability. Among the FuNPs tested, FuNP1 induced the most significant oxidative stress in bacteria, likely due to its higher and faster NO release compared to the other nanoparticles (Figure 2d). Notably, PMB/FuNPs caused significant oxidative stress in Gram‐negative bacteria, resulting in a marked increase in fluorescence intensity. This synergistic effect can be attributed to PMB's ability to increase membrane permeability at sub‐MIC levels, allowing more NO released from FuNPs to enter the bacterial cells efficiently. The increased membrane permeability facilitates greater penetration of NO into the bacterial cytoplasm, leading to higher intracellular NO concentrations. This observation aligns with our previous studies, which suggest that the perturbation of Gram‐negative bacterial membranes by low concentrations of PMB facilitates the entry of other antimicrobials, enhancing their bactericidal effects.**^[^** ^16^, ^17^ ^]^

### 2.5 Neutron Reflectometry Reveals FuNP‐Membrane Interaction

To elucidate the intricate antibacterial mechanisms of PMB/FuNPs, our investigation employed neutron reflectometry to probe the ångström‐level interactions among FuNP1, PMB and PMB/FuNP1, both prior to and after NO release, against specially engineered model membranes. These membranes were intricately designed to simulate the unique structural configurations of bacterial cell membranes,^[^ ^16^, ^17^ ^]^ incorporating essential components such as LPS, lipid A (a principal virulence factor of LPS) and phospholipids. This design ensures an accurate representation of the membrane's fundamental components, allowing for a detailed investigation into how FuNPs interact with and potentially disrupt bacterial cellular structures. In our simulation of a combinatorial therapeutic modality, model membranes were initially exposed to FuNP1, either as‐is or after NO release, before being subjected to PMB exposure. The application of various isotopic contrast solutions (Table S15, Supporting Information) facilitated the acquisition of significant insights, as indicated by alterations in the reflectivity profiles of LPS, lipid A and phospholipid bilayer for both pre‐ (**Figure** **5**a‐c,g‐l; Figures S9–S12, Supporting Information) and post‐NO release scenarios (Figure 5d‐f; Figures S13–S15, Supporting Information).

Figure 5Mechanistic study of PMB supplied together with FuNPs (PMB/FuNPs) by neutron reflectometry. Reflectivity profiles (symbols) and fits (lines) of a) LPS: 1,2‐dipalmitoyl‐d_62_‐sn‐glycero‐3‐phosphocholine (d‐DPPC) outer membrane bilayer. b) lipid A: d‐DPPC outer membrane bilayer. c) inner membrane bilayer (black curves). Bilayers were initially treated with FuNP1 (128 µg mL^−1^, red curve) followed by PMB (4 µg mL^−1^, blue curve) in D_2_O buffer. d–f) Identical bilayers to (a–c) initially treated with PMB (4 µg mL^−1^, red curve) followed by FuNP1 (128 µg mL^−1^, blue curve) in D_2_O buffer. The inserted cartoons depict the bilayer structure after PMB/FuNP1 combination treatment. All data are expressed as median ± s.d. (indicated by error bars), based on values obtained from three isotopic contrasts, i.e., D_2_O, CMSi (contrast‐matched silicon) and H_2_O. g) The fitted volume fraction of bilayer (a) before and after successive treatment with FuNP1 and PMB. h) The fitted thickness of bilayer (a) before (dark bar) and after successive treatments with FuNP1 (red bar) and PMB (blue bar). i) The fitted volume fraction of bilayer (b) before and after successive treatments with FuNP1 and PMB. j) The fitted thickness of bilayer (b) before (dark bar) and after successive treatment with FuNP1 (red bar) and PMB (blue bar). k) The fitted volume fraction of bilayer (c) before and after successive treatments with FuNP1 and PMB. l) The fitted thickness of bilayer (f) before (dark bar) and after successive treatments with FuNP1 (red bar) and PMB (blue bar). m) Proposed multimodal membrane interaction mechanism of PMB supplied together with FuNPs. 1) First, FuNPs approach the bacteria and bind to the polysaccharide from the cell surface. 2) Second, PMB freely destabilizes the outer membrane and assists the FuNPs to reach the phospholipid layer. 3) Third, PMB/FuNPs destroy the phospholipid bilayer, meanwhile, L‐cysteine from the bacterial biosynthesis triggers the NO releasing from FuNPs. 4) Phospholipid bilayer is then destroyed by PMB/FuNPs synergistic effect, and the released NO from FuNPs transports into cytoplasm. 5) Gaseous NO further performs its inherent antimicrobial efficacy in cytoplasm through a series of mechanisms. a) NO is released from FuNPs through the interaction with L‐cysteine from the bacterial biosynthesis. b) Gaseous NO is not able to penetrate the bacterial outer membrane.

**Figure:**
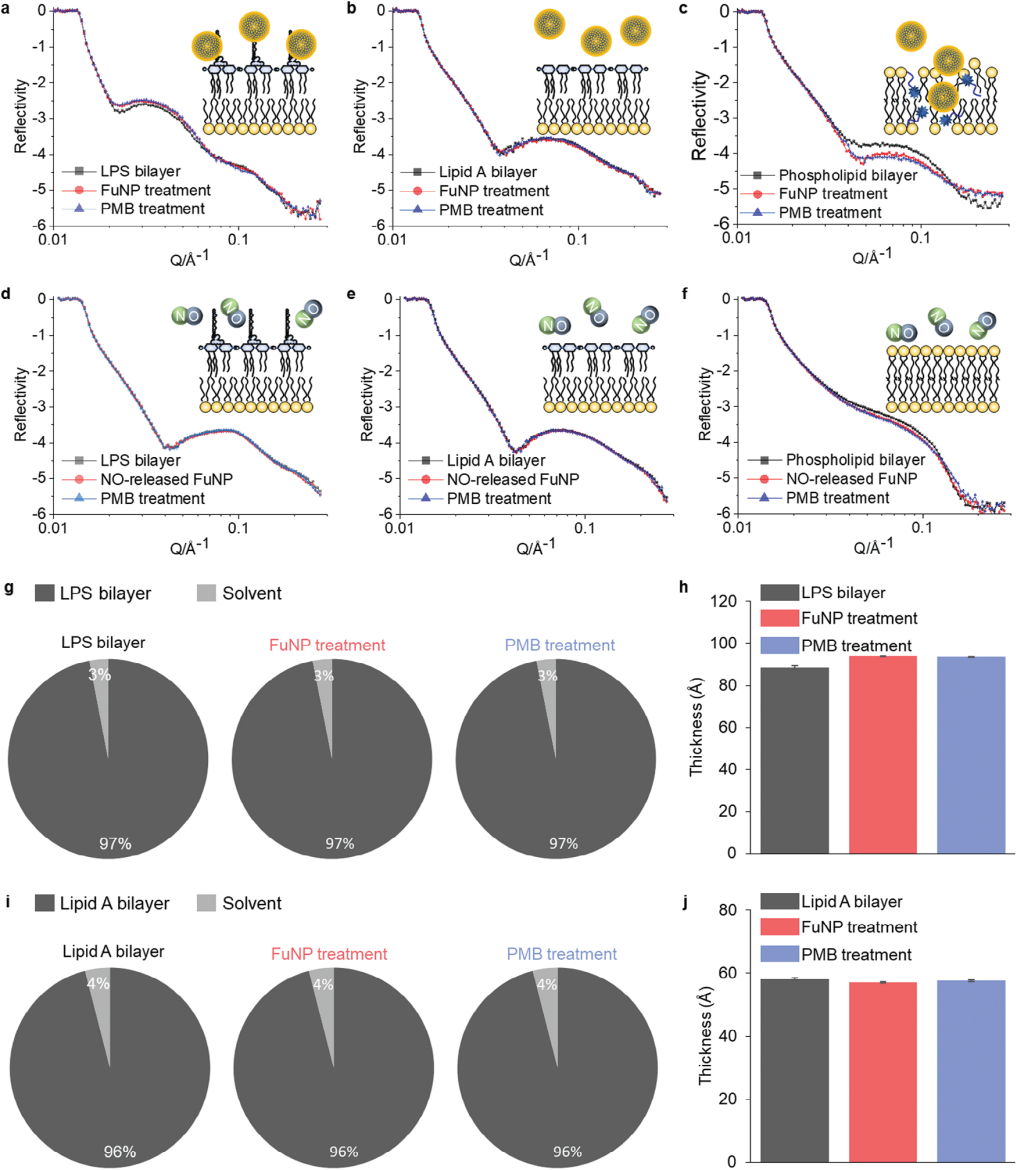

**Figure:**
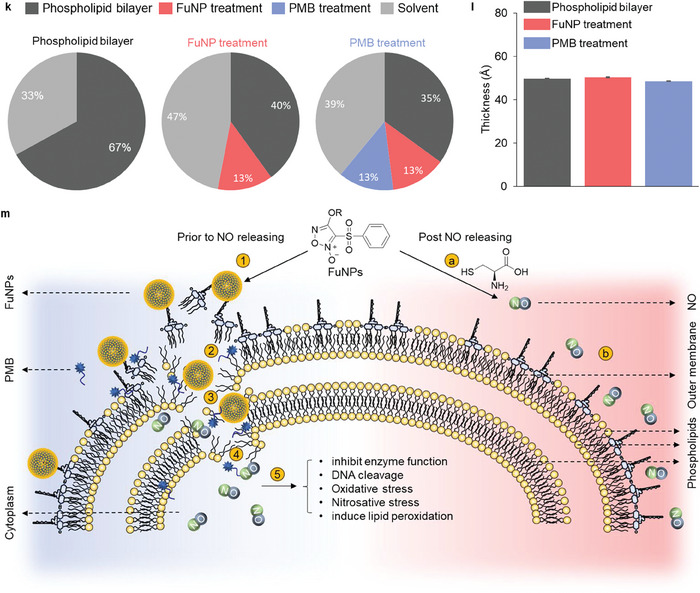

#### 2.5.1 FuNP1 Significantly Alters the LPS Layer

Analysis of FuNP1's interaction with the LPS bilayer revealed significant alterations, evidenced by shifts in specular reflectivity intensities (Figure 5a, black to red curves). The degree of this interaction was quantified by the sustained volume fraction (Figure 5g; Figure S9 and Table S15, Supporting Information), coupled with an observed increase in the bilayer's thickness from an average of 88.4 ± 0.8 to 93.7 ± 0.1 Å (Figure 5h; Table S16, Supporting Information), suggesting FuNP1' adherence to the LPS surface without bilayer penetration (Figure 5m①). Conversely, post‐NO release, FuNP1 failed to induce notable changes in the LPS membrane's reflectivity profiles (Figure 5d and Figure 5mⓐ‐ⓑ; Figure S13, Supporting Information). The observed behavior sheds light on the lack of effectiveness seen with FuNP1 monotherapy in in vitro antibacterial assays, as well as the reduced antibacterial activity of NO‐released FuNP1 when supplied together with PMB (Table S12, Supporting Information). The diminished antibacterial activity post‐NO release suggests a disruption in FuNP1's self‐assembly structure, crucial for its NO delivery mechanism and overall antibacterial function, highlighting the critical role that the self‐assembly of FuNP1 plays in their functionality.

#### 2.5.2 FuNP1 does not Bind to Lipid A

To ascertain whether FuNP1 interactions predominantly occur with lipid A, the latter was initially subjected to FuNP1 treatment (Figure 5b; Figure S10, Supporting Information). The absence of any discernible changes in reflectivity profiles, volume fractions, and bilayer thickness post‐treatment suggests a minimal engagement with lipid A (Figure 5i‐j; Table S17, Supporting Information). This pattern persisted following NO release (Figure 5e,i‐j; Figure S14, Supporting Information), reinforcing the notion that FuNP1 interactions target the polysaccharide components of LPS rather than lipid constituents. This observation is in line with the ToF‐SIMS results (Figure 4d), demonstrating the PMB/FuNP1's interaction with polysaccharides, leading to potential membrane damage. Furthermore, this observation implies that released NO does not disrupt the lipid A barrier, whereas the PMB/FuNP1 significantly inhibits bacterial growth, implying that PMB must first destabilize the lipid A barrier to facilitate FuNP1 entry, thereby inducing a synergistic antibacterial effect (Figure 5m③). Extended neutron experiments, involving initial PMB treatment of the lipid A bilayer followed by FuNP1 application, supported this hypothesis by demonstrating noticeable changes in reflectivity profiles (Figure S11, Supporting Information), which supports our assumptions.

#### 2.5.3 FuNP1 Disrupts Phospholipids

The phospholipid bilayer experienced significant disruption following FuNP1 treatment, as indicated by marked shifts in neutron reflectivity profiles (Figure 5c; Figure S12, Supporting Information). Quantitative analysis revealed a substantial penetration of FuNP1 (13.0% ± 6.2%) into the phospholipid bilayer and a notable reduction (≈40%) in the volume fraction of the bilayer (Figure 5k). No significant change in bilayer thickness was observed (Figure 5l; Table S18, Supporting Information). Subsequent PMB treatment, simulating the PMB/FuNP1 therapy, further compromised the membrane, with 12.7% ± 4.5% of the PMB penetrating the bilayer (Figure 5m④; Table S18, Supporting Information), underscoring the synergistic effect of PMB and FuNP1. Noting that although NO‐released FuNP1 mildly interfered with the phospholipid bilayer, its combination with PMB did not exacerbate bilayer damage, as evidenced by the unchanged reflectivity profile post‐treatment (Figure 5f, red to blue curve; Figure S15, Supporting Information). Collectively, these findings confirm that FuNP1's initial disruption of the phospholipid bilayer, followed by its synergistic activity with PMB, is a key mechanism underlying the antimicrobial efficacy of the PMB/FuNP1. Due to the inherent biosynthesis of L‐cysteine, the NO delivered by FuNP1 will continue its antimicrobial activity within the cytoplasm to further compromise the bacterial viability (Figure 5m⑤, Figure S8, Supporting Information).

## 3 Conclusion 

In summary, our study presents the pioneering development of self‐assembling furoxan derivatives into FuNPs, a novel type of nanoparticles capable of efficiently delivering NO to achieve bactericidal effects at low concentrations of NO. These FuNPs demonstrate notable stability under physiological conditions for at least 72 h, enhancing their potential for biological applications. When combined with PMB, the FuNPs effectively eliminate a range of Gram‐negative bacteria, such as *A. baumannii*, *P. aeruginosa*, *K. pneumoniae*, and *E. coli*, including strains with high resistance to PMB. Importantly, this combination markedly diminishes the likelihood of developing antimicrobial resistance to both PMB and FuNPs. Significant morphological alterations observed in all Gram‐negative bacteria tested post‐treatment with the PMB/FuNPs highlight the destructive impact on bacterial membranes. Our investigation into the action mechanism of FuNPs, supported by neutron reflectometry, unveiled a dual‐action mechanism. Before releasing NO, FuNPs specifically target Gram‐negative bacteria by binding to polysaccharides on the outer membrane, compromising the inner membrane's integrity in a synergistic action with PMB. Our work not only enriches the field of antimicrobial research but also establishes a groundbreaking use of the self‐assembly technique for NO release in fighting bacterial infections. Future studies will focus on in vivo experimentation to validate the therapeutic potential and safety of FuNPs in animal models, further advancing their clinical applicability.

## 4 Experimental Section

### Characterization

The characterization techniques were conducted in accordance with literature methods.^[^ ^16^, ^17^ ^]^ Briefly, NMR spectra were obtained with a Bruker Advance III 400 (9.4 T) spectrometer using a 5 mm broadband auto tunable probe featuring Z‐gradients and equipped with a BACS 60 tube autosampler, with chemical shifts (δ) reported in ppm relative to DMSO‐d6 (δ 2.5). FuNPs were stained with uranyl acetate for 1 min and the TEM images were captured using a JEOL JEM‐1400PLUS TEM at 80 keV with a bottom‐mounted CMOS camera. SEM analysis was conducted with a FEI Nova NanoSEM 450 at 2 kV in immersion mode. High‐resolution and confocal optical imaging were performed with an Olympus FV3000 Confocal Laser Scanning Microscope, while probe sonication utilized a QSonica Q125 sonicator at ambient temperature. Dynamic light scattering (DLS) measurements were taken at 37 °C using a Malvern Zetasizer Nano S, and fluorescence was measured with a Victor Nivo Multimode Plate Reader at room temperature.

### Synthesis of Furoxan‐1

*tert*‐butyl (2‐(2‐hydroxyethoxy) ethyl) carbamate (670 mg, 3.27 mmol, 1.5 equiv) and bis(phenylsulfonyl)furoxan (800 mg, 2.18 mmol, 1.0 equiv) were dissolved in 15 mL of dichloromethane, followed by the addition of 1,8‐diazabicyclo(5.4.0) undec‐7‐ene (DBU, 994 mg, 6.54 mmol, 3 equiv). The reaction was stirred at room temperature for 16 h, concentrated and subjected to flash column purification on silica gel with petroleum benzine and ethyl acetate (2:1, v/v) as eluent to give compound **III‐a**. The adduct was subsequently dissolved in 10 mL of dichloromethane and trifluoroacetic acid (1 mL) was added to the reaction mixture. The reaction was stirred at room temperature for 4 h and concentrated to give the corresponding compound furoxan‐1 (600 mg, 64% yield) without further purification. ^1^H NMR (400 MHz, DMSO‐d_6_) δ 8.02 (dd, *J* = 8.6, 1.2 Hz, 2H), 7.95–7.80 (m, 4H), 7.78–7.73 (m, 2H), 4.62–4.51 (m, 2H), 3.91–3.81 (m, 2H), 3.70 (t, *J* = 5.3 Hz, 2H), 3.03 (h, *J* = 5.8 Hz, 2H); ^13^C NMR (101 MHz, DMSO‐d_6_) δ 159.0, 158.4, 158.1, 137.1, 136.2, 130.0, 128.4, 110.6, 70.7, 67.7, 66.8, 38.6.

### Synthesis of Furoxan‐2

*tert*‐butyl (5‐hydroxypentyl) carbamate (663 mg, 3.27 mmol, 1.5 equiv) and bis(phenylsulfonyl)furoxan (800 mg, 2.18 mmol, 1.0 equiv) were dissolved in 15 mL of dichloromethane, followed by the addition of DBU (994 mg, 6.54 mmol, 3 equiv). The reaction was stirred at room temperature for 16 h, concentrated and subjected to flash column purification on silica gel with petroleum benzine and ethyl acetate (2:1, v/v) as eluent to give compound **III‐b**. The adduct was subsequently dissolved in 10 mL of dichloromethane and trifluoroacetic acid (1 mL) was added to the reaction mixture. The reaction was stirred at room temperature for 4 h and concentrated to give the corresponding compound furoxan‐2 (520 mg, 56% yield) without further purification. ^1^H NMR (600 MHz, DMSO‐d_6_) δ 8.01 (dd, *J* = 8.5, 1.2 Hz, 2H), 7.93–7.89 (m, 1H), 7.78–7.69 (m, 5H), 4.40 (t, *J* = 6.4 Hz, 2H), 2.84–2.78 (m, 2H), 1.81–1.75 (m, 2H), 1.59 (p, *J* = 7.6 Hz, 2H), 1.41 (q, *J* = 7.7 Hz, 2H); ^13^C NMR (151 MHz, DMSO‐d_6_) δ 158.9, 158.3, 158.1, 137.2, 136.2, 130.0, 128.3, 110.5, 71.2, 38.6, 27.3, 26.4, 21.9.

### Synthesis of Furoxan‐3

*tert*‐butyl (5‐hydroxy‐4,4‐dimethylpentyl) carbamate (661 mg, 2.86 mmol, 1.5 equiv) and bis(phenylsulfonyl)furoxan (700 mg, 1.91 mmol, 1.0 equiv) were dissolved in 15 mL of dichloromethane, followed by the addition of DBU (871 mg, 5.73 mmol, 3 equiv). The reaction was stirred at room temperature for 16 h, concentrated and subjected to flash column purification on silica gel with petroleum benzine and ethyl acetate (4:1, v/v) as eluent to give compound **III‐c**. The adduct was subsequently dissolved in 10 mL of dichloromethane and trifluoroacetic acid (1 mL) was added to the reaction mixture. The reaction was stirred at room temperature for 4 h and concentrated to give the corresponding compound furoxan‐3 (450 mg, 52% yield) without further purification. ^1^H NMR (400 MHz, DMSO‐d_6_) δ 8.01 (d, *J* = 8.3 Hz, 2H), 7.91 (t, *J* = 7.5 Hz, 1H), 7.80–7.68 (m, 5H), 4.10 (s, 2H), 2.77 (q, *J* = 6.8 Hz, 2H), 1.52 (q, *J* = 7.8 Hz, 2H), 1.37–1.29 (m, 2H), 0.96 (s, 6H); ^13^C NMR (101 MHz, DMSO‐d_6_) δ 159.1, 158.4, 158.0, 137.3, 136.2, 130.1, 128.2, 110.3, 78.3, 34.6, 33.8, 23.4, 21.7.

### Preparation of FuNPs

The self‐assembly method for synthesizing furoxan‐1 nanoparticles (FuNP1) was demonstrated, with the process being adaptable for furoxan‐2 and 3 by substituting furoxan‐1 accordingly. Initially, 10 mg of furoxan‐1 and 10 wt.% of F127 were dissolved in 5 mL chloroform within a glass vial. The mixture was thoroughly stirred for 1 min at room temperature to ensure complete dissolution. The chloroform was then evaporated under a gentle stream of nitrogen gas until a thin film of furoxan‐1 formed at the bottom of the vial. This film was further dried in a desiccator under vacuum at room temperature for a minimum of 24 h to remove any residual solvent. Subsequently, specific volumes of 1X PBS (pH 7.4) were added to achieve a 1 mg mL^−1^ furoxan‐1 concentration. The PBS was added gradually while the solution was gently stirred to avoid clumping. The resulting mixture was subjected to probe sonication using a Qsonica sonicator (Adelab Scientific) for 5 min. The sonication was performed with 5‐s. on and 5‐s off cycles at 50% amplification (125‐Watt, 20 kHz). This sonication process was conducted in an ice bath to prevent overheating, maintaining the temperature below 25 °C.

### Quantification of NO and NO_x_ Released from FuNPs^[^ ^14a,c^ ^]^

NO release was quantified using a 2,3‐diaminonaphthalene (DAN)‐based chemical assay, which relies on the reaction of NO with oxygen to form dinitrogen trioxide (N₂O₃), subsequently reacting with nonfluorescent DAN to form the highly fluorescent 2,3‐naphthotriazole (NAT). Briefly, 50 µg of DAN was dissolved in 1 mL of 0.62 M HCl to prepare a 0.31 mM DAN solution. This solution (10 µL) was mixed with 100 µL of either NaNO₂ solution (0–10 µm) or the sample solution (50 µm) and incubated in the dark at room temperature for 10–15 min. Then, 5 µL of 3 m NaOH was added to the reaction mixture. The resulting solution was diluted with 4 mL of water, and fluorescence was measured with an excitation wavelength of 365 nm and an emission wavelength of 450 nm. A calibration curve (0–10 µm) was constructed with NaNO₂ concentration on the X‐axis and fluorescence intensity on the Y‐axis, which was then used to determine the NO₂ concentration in the sample solutions. Griess assay was used to quantify total NO_x_ release in transparent 96‐well polystyrene plates. Briefly, the FuNPs (0.25 mm, 82.5 µg mL^−1^) were dissolved in 1X PBS, followed by the addition of L‐cysteine (2.5 mm, 302.9 µg mL^−1^). The mixture was incubated at 37 °C for 24 h. Aliquots of 50 µL were taken at 0.5, 1, 2, 4, 6, 8, 12, and 24 h, then incubated for 15 min at room temperature with 50 µL of Griess reagent A (1.0% sulfanilamide, 5.0% H₃PO₄ in dH₂O) and Griess reagent B (0.1% (N‐1‐naphthyl) ethylenediamine dihydrochloride in dH₂O) (G4410, Sigma–Aldrich). The UV absorbance at 540 nm was measured using a microplate spectrophotometer and calibrated using a standard curve constructed with 0–100 µm NaNO₂ in PBS (pH 7.4) to yield nitrite concentration.

### Minimum Inhibitory Concentration (MIC)

A colony‐forming unit (CFU) measurement was utilized to determine the viable bacteria count in a sample. Initially, Mueller–Hinton Broth (MHB with 20–25 mg L^−1^ Ca^2+^ and 10–12.5 mg L^−1^ Mg^2+^) was used to create twofold dilutions of an antimicrobial agent. A 96‐well microtiter plate was then prepared with 100 µL of these antimicrobial dilutions. Bacterial suspensions were adjusted to ≈10^8^ CFU mL^−1^ and subsequently diluted 1:100 in MHB to reach a concentration of ≈10^6^ CFU mL^−1^ of the viable bacteria. Within 15 min, 100 µL of antimicrobials were mixed using a vortex, and then added to the microtiter plate wells, achieving a final inoculum concentration of ≈5 × 10^5^ CFU mL^−1^. The MIC was identified after a 16–20‐h incubation at 37 °C. These procedures were replicated three times (n = 3) for consistency.

### Fractional Inhibitory Concentration (FIC)

FIC measures the efficacy of antibiotic combinations versus their individual effects, using the microdilution checkerboard method aligned with CLSI standards.^[^ ^18^ ^]^ This process involves PMB at 1/4 × MIC in combination with FuNPs. Solutions were added to a 96‐well plate, followed by a bacterial suspension of *A. baumannii*, *P. aeruginosa*, *K. pneumoniae*, or *E. coli*, aiming for a final concentration of ≈5 × 10^5^ CFU mL^−1^. After 16–20 h of incubation at 37 °C, FIC values were assessed. The FIC index (FICI) calculates the lowest concentration at which antimicrobial agents completely inhibit growth, using a formula: FICI = (FIC_A1_/MIC_A1_) + (FIC_A2_/MIC_A2_), where A1 = PMB and A2 = FuNPs, to assess synergistic (FICI ≤0.5), indifferent (0.5 < FICI < 4), or antagonistic (FICI ≥4) interactions.^[^ ^19^ ^]^ These procedures were replicated three times (n = 3) for consistency.

### Growth Curve Assay^[^ ^20^ ^]^

An individual colony was grown in 5 mL of MHB broth at 37 °C overnight. The resulting stationary‐phase cultures were diluted in fresh MHB to an OD_600_ nm of 0.2, serving as the initial cultures for susceptibility tests. These tests were done in triplicate on a 96‐well plate, with each well containing 180 µL of the initial culture, PMB at 1/4 × MIC, and FuNP concentrations from 32 to 128 µg mL^−1^. The total volume in each well was topped up to 200 µL using MHB broth. The plate, sealed with a transparent lid, was then incubated at 37 °C in a VICTOR Nivo plate reader. Absorbance at OD600 nm was recorded every 60 min over a 24‐h period. The entire procedure was repeated three times (n = 3) for consistency.

### Resistance Studies

Experimental evolution was performed based on a previously established evolution experiment with minor modification.^[^ ^21^ ^]^ The bacterial culture was incubated and on day 1 the MIC concentrations of PMB, FuNPs or PMB/FuNPs were determined to be the lowest concentration in which no growth was observed. The 1/2 × MIC concentration culture was used to inoculate a second serially diluted series of antimicrobial compound and incubated for 24 h. The next day the MIC (which may remain the same or increase) was determined and 2 µL of the 1/2 × MIC concentration culture from the latest passage was used to inoculate a new series of 198 µL of diluted antimicrobial compounds. The process continues for up to 8 days.

### Cell Cytotoxicity Assay

Cell viability was assessed using the MTT [3‐(4,5‐dimethylthiazol‐2‐yl)‐2,5‐diphenyltetrazolium bromide] assay. Human HeLa cells were seeded into tissue culture‐treated 96‐well plates at a density of 5 × 10^3^ cells per well and incubated overnight at 37 °C with 5% CO₂. The cells were then treated with Dulbecco's Modified Eagle's Medium supplemented with 10% fetal bovine serum, containing different concentrations of PMB, FuNPs, and PMB/FuNPs for various time points (15 min, 30 min, 1 h, 2 h, 4 h, 6 h, 24 h, 48 h, and 72 h). After treatment, the medium was replaced with fresh medium containing 0.5 mg mL^−1^ MTT and incubated at 37 °C for 4 h. Subsequently, the medium was removed and replaced with DMSO, followed by an overnight incubation at 37 °C to dissolve the formazan crystals. Absorbance was measured at 570 nm using a VICTOR Nivo Plate Reader.

### ToF‐SIMS

ToF‐SIMS data were acquired with a TOF.SIMS 5 instrument (IONTOF GmbH, Germany). Three sample replicates of each treatment type (control, FuNP1, PMB and PMB/FuNP1), were prepared using the procedures identical to SEM sample preparation.^[^ ^17^ ^]^ A 30 keV Bi_3_^+^ primary ion was used to collect five replicate spectra from each sample in positive ion mode, averaging 128 × 128 pixels over a 250 × 250 µm area. Each spectrum was collected with a cycle time of 100 µs, with one shot per frame and one frame over 20 scans. A surface potential of −60 V, an electron flood gun and argon flooding (raising the vacuum level in the analysis chamber to 1 × 10^−6^ mbar) were used for charge compensation. Spectra were calibrated using an organic peak list (C^+^, CH^+^ CH_2_^+^, C_2_H_2_^+^, C_3_H_2_^+^, C_4_H_3_^+^, C_5_H_3_^+^, C_6_H_5_^+^, C_7_H_7_^+^), then exported to ASCII text files and plotted in MATLAB v2023b (after interpolation to a common m/z range and normalization to total ion dose), using the shadedErrorBar toolbox.^[^ ^22^ ^]^

### Membrane Permeability

2′,7′‐Dichlorodihydrofluorescein diacetate (DCFH‐DA) was used to detect membrane permeability in bacterial cells. Bacterial cultures were grown to an optical density (McFarland 0.5), harvested by centrifugation at 5000 x g for 5 min, and washed twice with 1X PBS. The bacterial pellet was resuspended in 1X PBS to a final concentration of 1 × 10^6^ CFU mL^−1^. DCFH‐DA was added to the suspension to a final concentration of 5 µm and incubated at 37 °C for 45 min in the dark with shaking at 100 rpm. Treatments were added to the bacterial suspension at the desired concentrations, and the mixture was incubated at 37 °C with shaking at 200 rpm. Controls included untreated cells (negative control), cells treated with DCFH‐DA only (positive control), cells treated with treatments and DCFH‐DA (experimental samples), and wells with PBS and DCFH‐DA only to assess background fluorescence. Fluorescence intensity was measured at 15‐min intervals for 24 h using a microplate reader with excitation at 485 nm and emission at 530 nm. Fluorescence intensity of treated samples was normalized to the negative control (untreated bacteria), and the fluorescence intensity of treated samples was compared to the positive control (bacteria treated with DCFH‐DA only) to assess membrane permeability. All steps involving DCFH‐DA were performed in the dark or under dim light to prevent photo‐oxidation of the probe. Fluorescence measurements were performed in triplicate.

### Gram‐Negative Membrane Bilayer Formation for Neutron Reflectometry

The model membrane was deposited on a SiO_2_ surface using a custom Langmuir‐Blodgett trough from Nima Technology, UK, adhering to established Langmuir–Blodgett and Langmuir–Schaefer techniques.**^[^** ^16^ ^]^ Asymmetric bilayers mimicking Gram‐negative bacterial outer membrane were created using LPS. A symmetric bilayer mimicking inner membrane was created using a mixture of synthetic PG (16:0−18:1), PE (16:0−18:1) and CL (18:1) in a molar ratio of 17:80:3. Lipids, dissolved in chloroform, were spread onto a 5 mm CaCl_2_ water surface at 15 °C. The membrane model was transferred onto the SiO_2_ surface in two phases: the inner leaflet via Langmuir–Blodgett deposition and the outer leaflet using Langmuir–Schaefer methodology. The silicon wafer bearing the bilayer was secured in an aluminum holder with a silicon backing plate, featuring inlet and outlet tubes for HEPES buffer exchange (5 mm CaCl_2_, 150 mm NaCl, 10 mm HEPES, pH 7.4).

### Neutron Reflectometry Measurement^[^ ^16^ ^]^

The analysis was carried out on the Platypus and Spatz time‐of‐flight neutron reflectometers at ANSTO's 20MW OPAL research reactor in Sydney, Australia.**^[^** ^23^ ^]^ Each instrument employs a cold neutron spectrum. In a vertical (for Platypus) or horizontal (for Spatz) scattering geometry, neutron reflections from the sample were captured at two angles of incidence (0.85° for 300 s and 3.5° for 3600 s), covering a momentum transfer (Q) range from 0.01 Å^−1^ to 0.3 Å^−1^, where Q = 4π sin(θ)/λ, with θ as the incidence angle and λ as the wavelength.

The symmetric membrane bilayer's properties were examined using three isotopic contrasts for scattering length density (SLD): D_2_O (99.9%, ρ = 6.35 × 10^−6^ Å^−2^), CMSi (contrast‐matched silicon, 38% D_2_O: 62% H_2_O v/v; ρ = 2.07 × 10^−6^ Å^−2^) and H_2_O (ρ = −0.56 × 10^−6^ Å^−2^). A 7 mL HEPES buffer solution (pH/D 7.4) was circulated through the sample at 1.0 mL min^−1^ using a pump (Knauer GmbH, Berlin, Germany) for contrast exchange. The bilayer's initial characterization in buffered contrasts confirmed its formation. It was then exposed to 128 µg mL^−1^ FuNP1 for 4 h, followed by a D_2_O buffer wash to remove non‐specific bindings. Post‐treatment, the bilayer was re‐characterized under all three contrasts. Subsequently, a second treatment with PMB was applied to study the combinational treatment effects on the MRSA model membrane.

Data was reduced to Q versus reflectivity using the refnx data reduction routine on both instruments.**^[^** ^24^ ^]^ This routine adjusts for detector efficiency, converts time‐of‐flight data to wavelength for Q calculation, re‐bins data to match instrument resolution, and merges datasets from two incidence angles at their overlap to form a complete reflectivity profile. It also normalizes the data so that the critical edge corresponds to a reflectivity of one. The final reflectivity data was expressed in terms of momentum transfer, Q, defined as Q = 4π sinθ / λ, with θ as the angle of incidence and λ as the neutron wavelength.

### Neutron Reflectometry Data Analysis

The neutron profiles were analyzed using refnx software.^[^ ^24^ ^]^ Briefly, the bilayer was segmented into sublayers characterized by thickness and scattering length density (SLD) (Table S14, Supporting Information). Volume fractions of each component in these sublayers were calculated by fitting the same layer under different isotopic contrasts simultaneously. For layers comprising a chemical species *s* and water *w*, the SLD is expressed as *ρ_layer_* = *φρ_s_* + (1‐ *φ*) *ρ_w_*, where *ρ_s_* and *ρ_w_* are the SLD of two components, respectively, and *φ* is the volume fraction of chemical species *s* in the layer. Considering the higher hydration in lipid headgroup regions due to their hydrophilicity, volume fractions were primarily determined by the lipid tail region's combined volume fractions.^[^ ^25^ ^]^

### Statistical Analysis

GraphPad Prism (version 10.1.2) was used for statistical analysis. Student's t‐tests were performed as unpaired two‐tailed analyses. One‐way ANOVA was used to determine the statistical differences between FuNP formulations. A p‐value of < 0.05 was considered statistically significant for all analyses.

## Conflict of Interest

The authors declare no conflict of interest.

## Author contributions

X.L. designed the study, ran most experiments, analyzed the data, and prepared the manuscript with contributions from all the authors. L.Y. synthesized and characterized the compounds of the study. X.H. conducted cytotoxicity assay, stability test and bacterial resistance assay. W.G., S.E.B., and P.J.P ran the ToF‐SIMS experiment and analyzed the data. S.W. assisted with the neutron experiment. A.P.L.B. provided technical support for neutron experiments and revised the manuscript. B.W.M., J.S., Y.W., and H.Y.H. revised the manuscript. P.W.H.C. directed the synthesis and characterization of the compounds of the study and provided foundation support. H.‐H.S. designed the project, provided the foundation support, and modified the structure of the manuscript. All authors were involved in discussing the results.

## Supporting information



Supporting Information

## Data Availability

The data that support the findings of this study are available in the supplementary material of this article.
