# Supplementary material for: Enhanced Nitric Oxide Delivery Through Self‐Assembling Nanoparticles for Eradicating Gram‐Negative Bacteria
Source: Adv Healthc Mater. 2024 Sep 12;13(32):2403046. doi: 10.1002/adhm.202403046 (PMC11670277; doi:10.1002/adhm.202403046)
Supplement: Supplementary file 1 — Supporting Information [file ADHM-13-0-s001.pdf]

ADVANCED HEALTHCARE MATERIALS

Supporting Information

for *Adv. Healthcare Mater.*, DOI 10.1002/adhm.202403046

Enhanced Nitric Oxide Delivery Through Self-Assembling Nanoparticles for Eradicating Gram-Negative Bacteria

Xiangfeng Lai, Lei Yu, Xiangyi Huang, Wil Gardner, Sarah E. Bamford, Paul J. Pigram, Shuhong Wang, Anton P. Le Brun, Benjamin W. Muir, Jiangning Song, Yajun Wang, Hsien-Yi Hsu, Philip Wai Hong Chan and Hsin-Hui Shen**

Supporting Information

Enhanced Nitric Oxide Delivery Through Self-Assembling Nanoparticles for Eradicating Gram-Negative Bacteria

Xiangfeng Lai, Lei Yu, Xiangyi Huang, Wil Gardner, Sarah E. Bamford, Paul J. Pigram, Shuhong Wang, Anton P. Le Brun, Benjamin W. Muir, Jiangning Song, Yajun Wang, Hsien-Yi Hsu, Philip Wai Hong Chan, and Hsin-Hui Shen**

X. Lai, X. Huang, S. Wang, H.-H. Shen
Department of Materials Science and Engineering, Faculty of Engineering, Monash University, Clayton, Victoria 3800, Australia.
Email: Hsin-Hui.Shen@monash.edu

L. Yu, P. W. H. Chan
School of Chemistry, Monash University, Clayton, Victoria 3800, Australia
Email: phil.chan@monash.edu

W. Gardner, S. E. Bamford, P. J. Pigram
Centre for Materials and Surface Science and Department of Mathematical and Physical Sciences, La Trobe University, Bundoora, 3086, Australia

A. P. Le Brun
Australian Centre for Neutron Scattering, Australian Nuclear Science and Technology Organisation, Locked Bag 2001, Kirrawee DC, New South Wales 2232, Australia.

B. W. Muir
CSIRO Manufacturing, Clayton, VIC 3168, Australia

Y. Wang
College of Chemistry & Materials Engineering, Wenzhou University, Wenzhou 325027, Zhejiang, China

H.-Y. Hsu
School of Energy and Environment & Department of Materials Science and Engineering, City University of Hong Kong, Kowloon Tong, Hong Kong, China; Shenzhen Research Institute of City University of Hong Kong, Shenzhen 518057, China.

J. Song, H.-H. Shen
Infection and Immunity Program, Monash Biomedicine Discovery Institute and Department of Microbiology, Monash University, Clayton, VIC 3800, Australia.
Email: Hsin-Hui.Shen@monash.edu

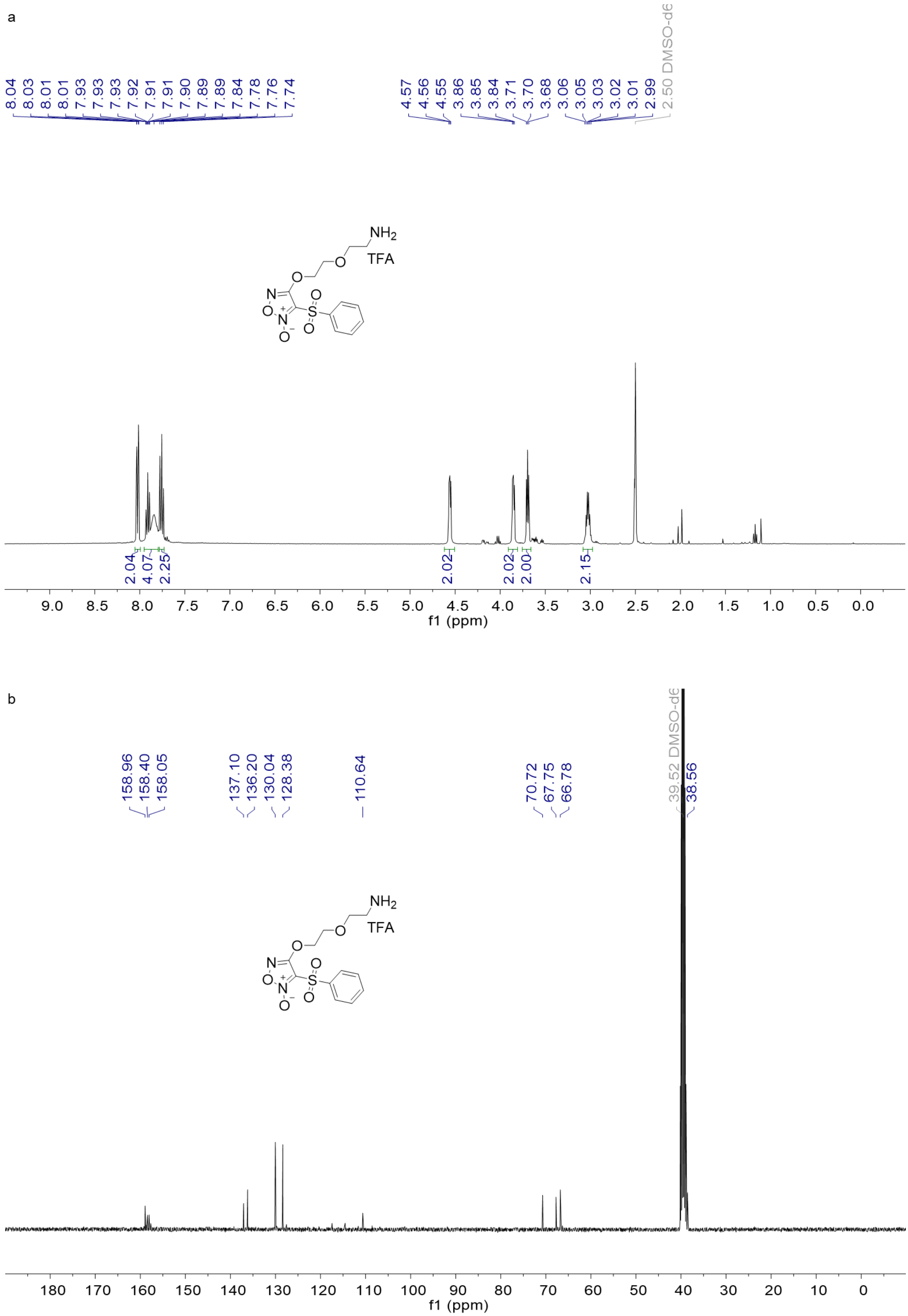

Figure S1. Characterization of FuNP1. (a) ^{1}H NMR spectrum of FuNP1. (b) ^{13}C NMR spectrum of FuNP1.

a

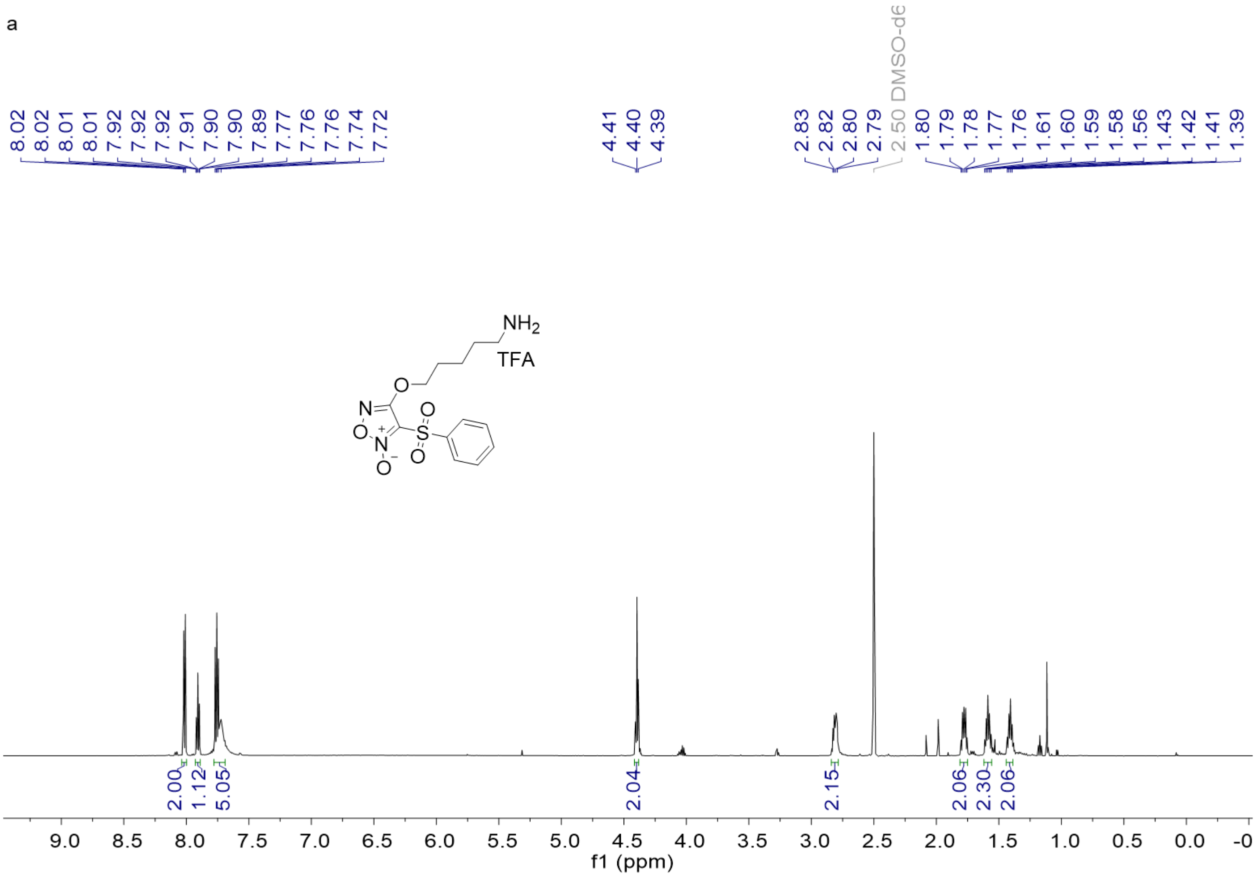

b

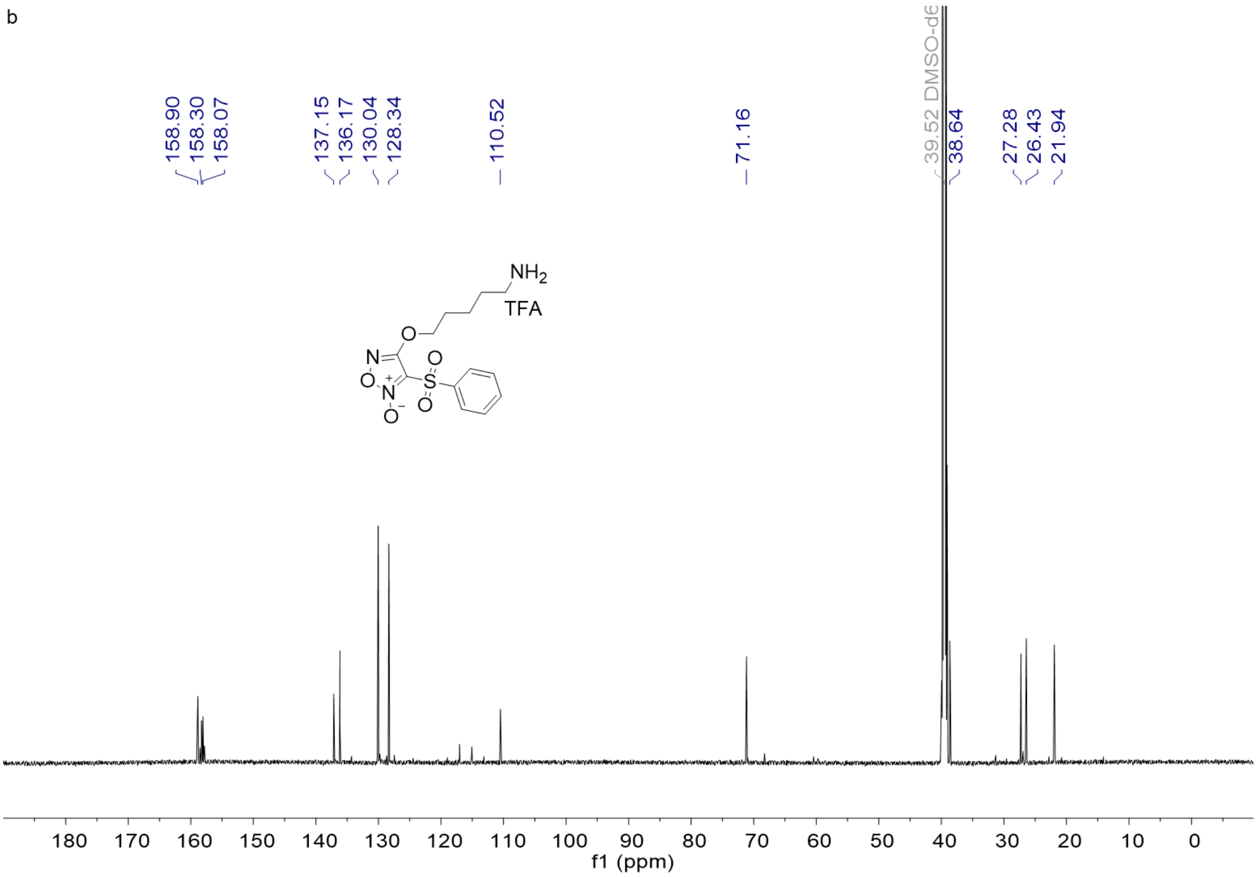

Figure S2. Characterization of FuNP2. (a) ^{1}H NMR spectrum of FuNP2. (b) ^{13}C NMR spectrum of FuNP2.

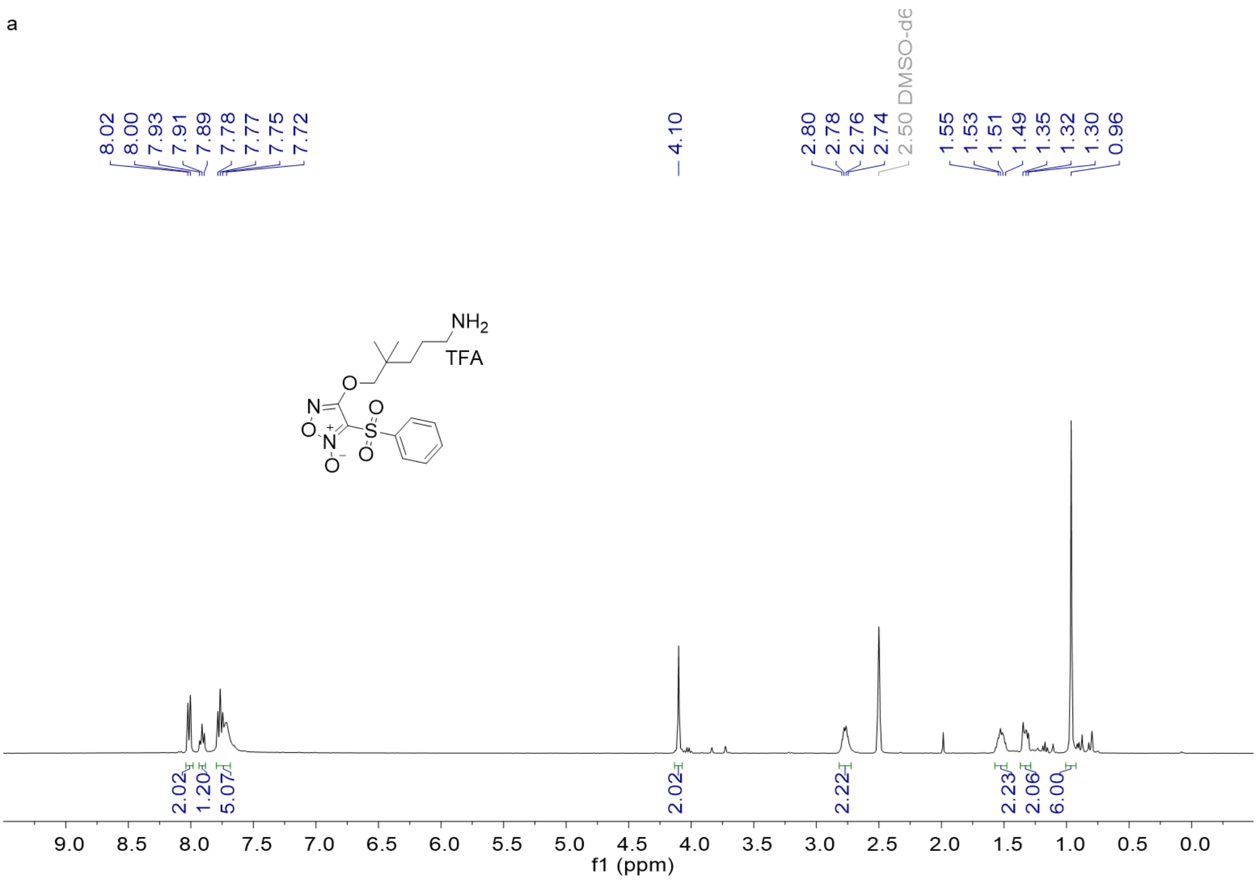

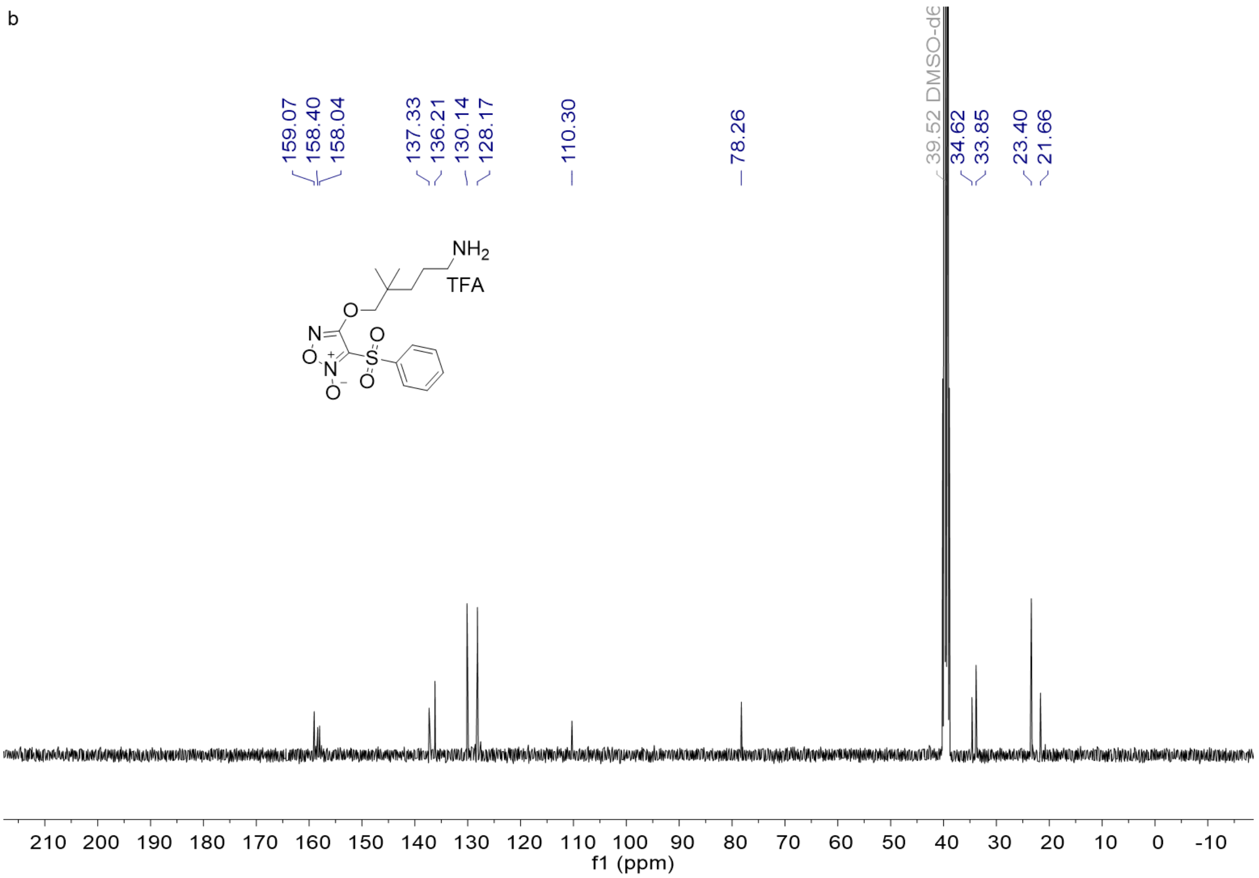

Figure S3. Characterization of FuNP3. (a) ^{1}H NMR spectrum of FuNP3. (b) ^{13}C NMR spectrum of FuNP3.

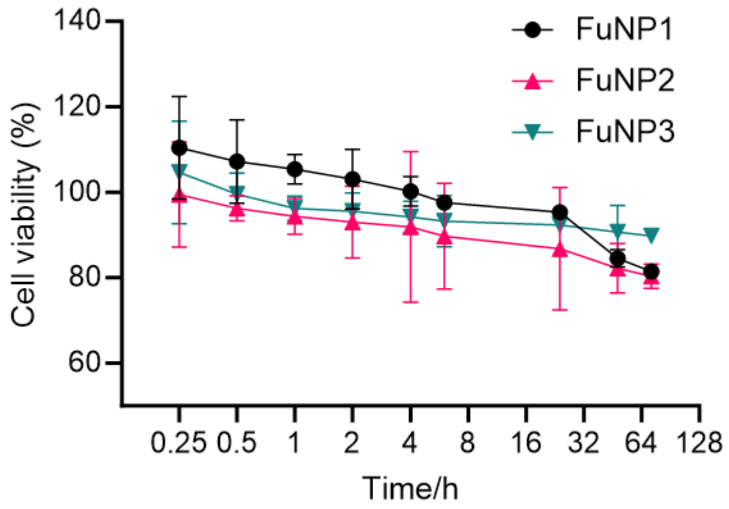

Figure S4. *In vitro* cell viability of human HeLa cells incubated in the presence of FuNPs (128 μg/mL) after incubation at different time points. Cell viability measurements are given relative to those of control samples (HeLa cells incubated in the absence of treatment). All Data are expressed as the mean ± S.D. All experiments were performed in triplicate (n=3).

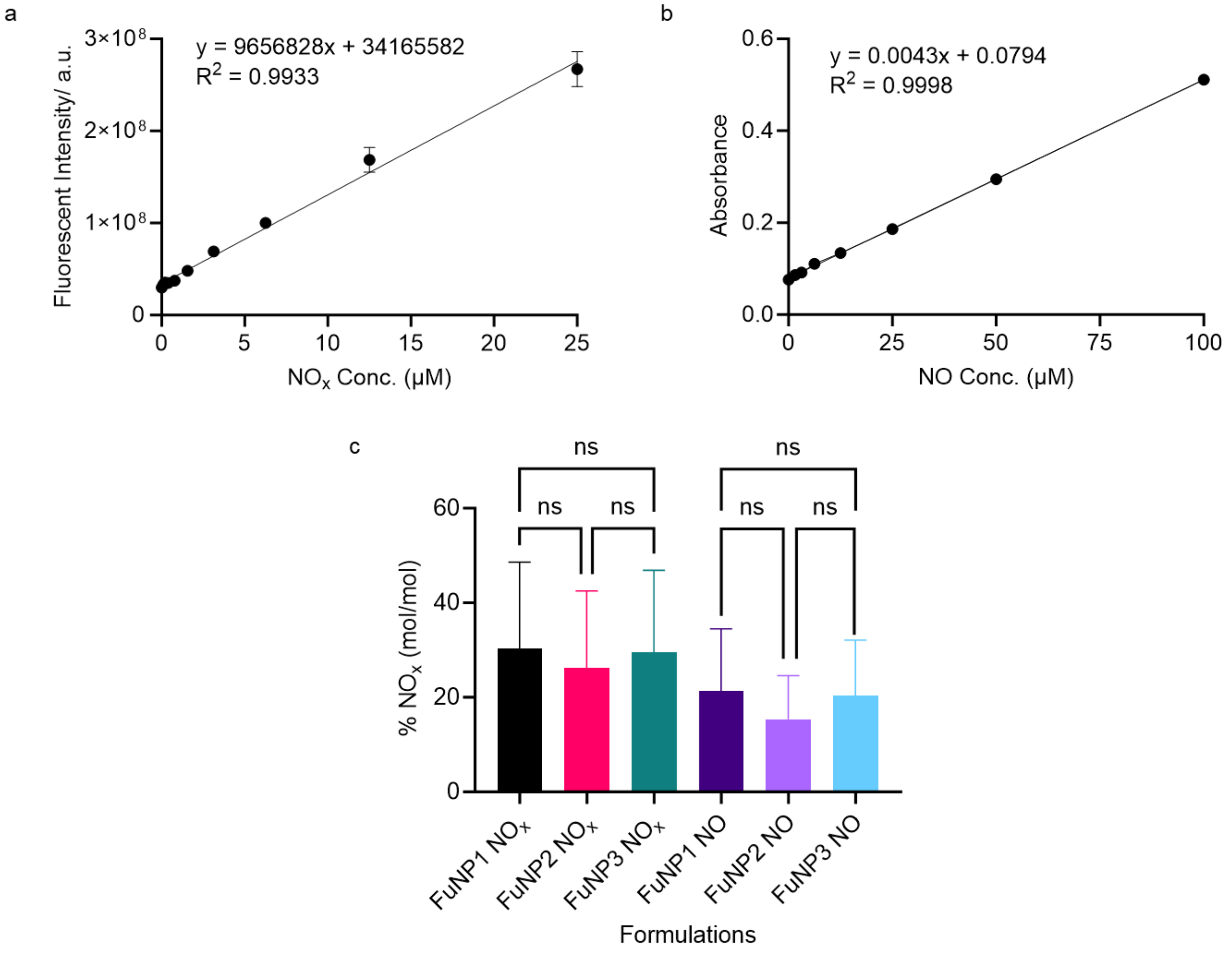

Figure S5. Analytical standard curves for the detection of NO_x (a) and NO (b) in phosphate buffer at pH 7.4 in the presence of L-cysteine. The x axis shows the NO_x and NO concentration whereas the y axis shows the fluorescence (Ex=365 mm, Em=450 nm) or absorbance units. (c) NO_x release kinetics (black circles) measured (n = 3) in the presence of L-cysteine and NO release kinetics (red circles) measured using the DAN-based assay of FuNPs over time at 37 °C (1X PBS, pH 7.4). The results are expressed as the percent (% mol/mol) of NO or NO_x released with respect to the quantity of parent furoxan compound. Statistical significance was determined by one-way ANOVA. ns, nonsignificant.

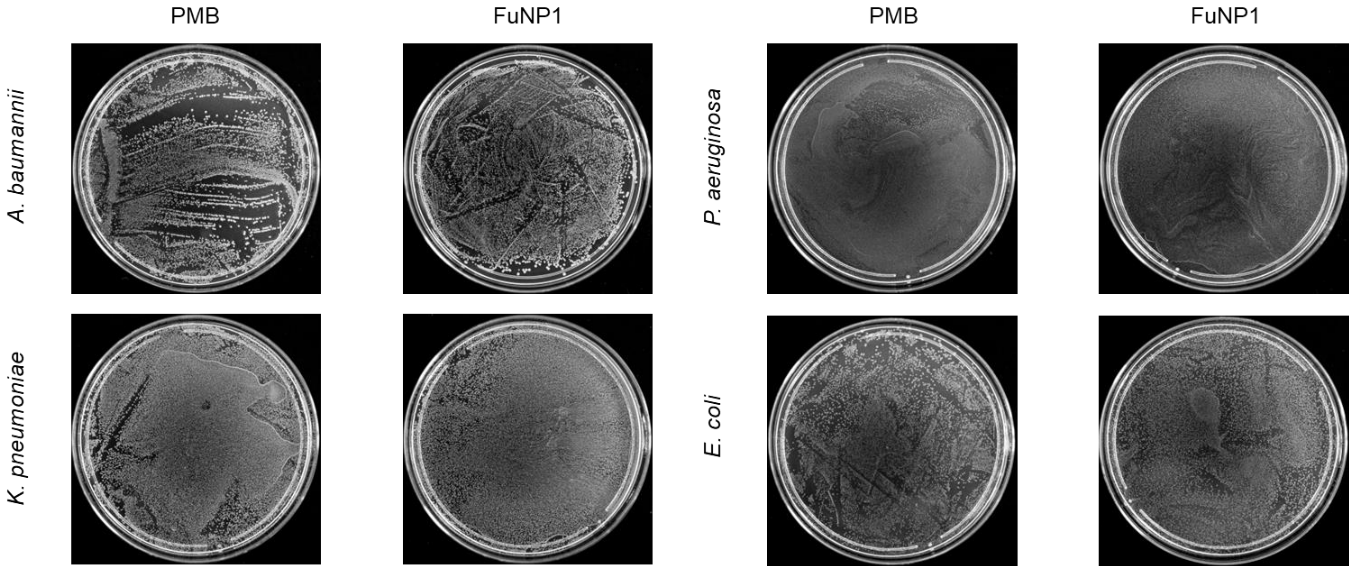

Figure S6. Representative images of *A. baumannii*, *P. aeruginosa*, *K. pneumoniae* and *E. coli* colonies cultured on the agar plates after various treatments for 24h.

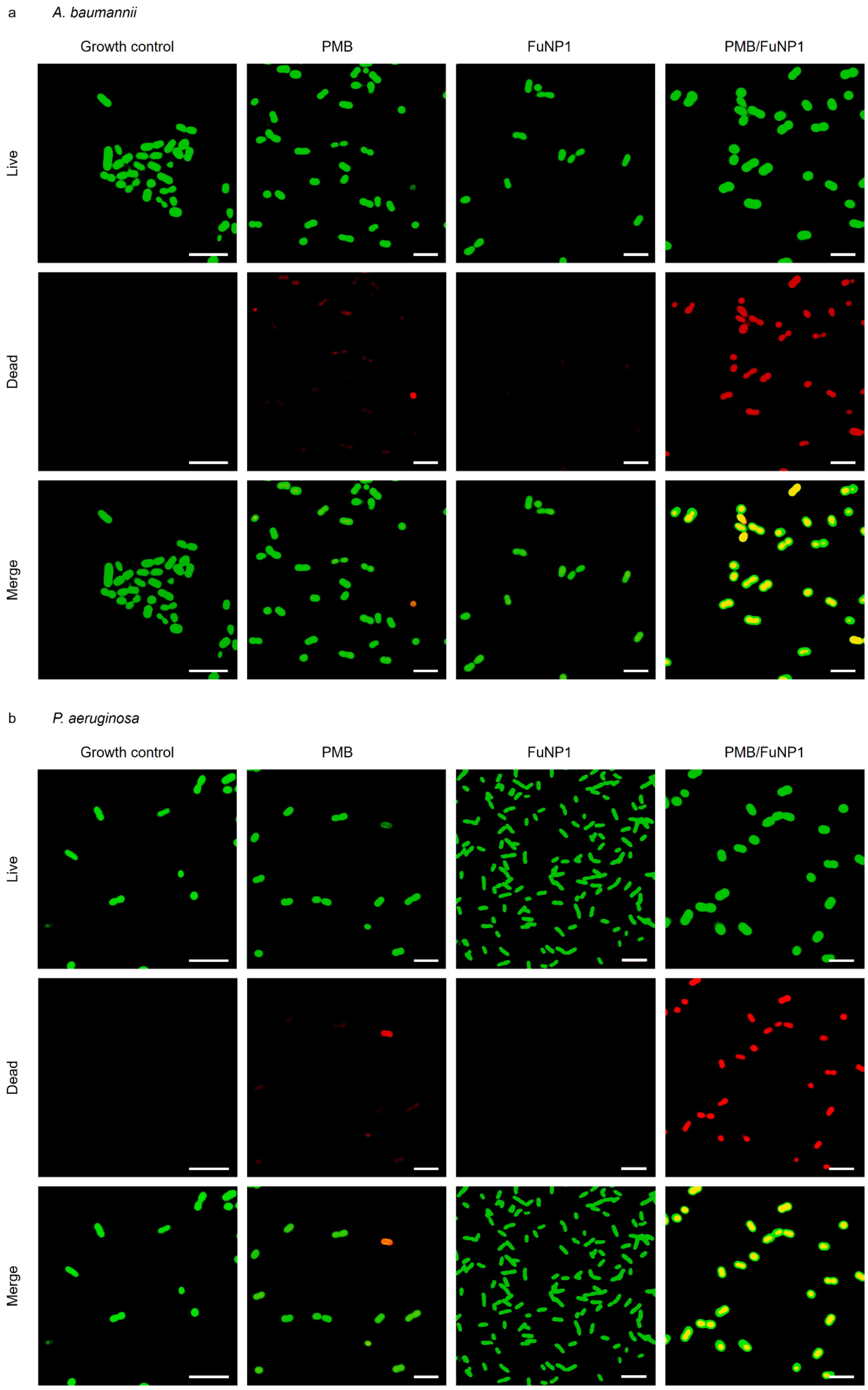
a
A. baumannii
Growth control
PMB
FuNP1
PMB/FuNP1
Live
Dead
Merge
b
P. aeruginosa
Growth control
PMB
FuNP1
PMB/FuNP1
Live
Dead
Merge

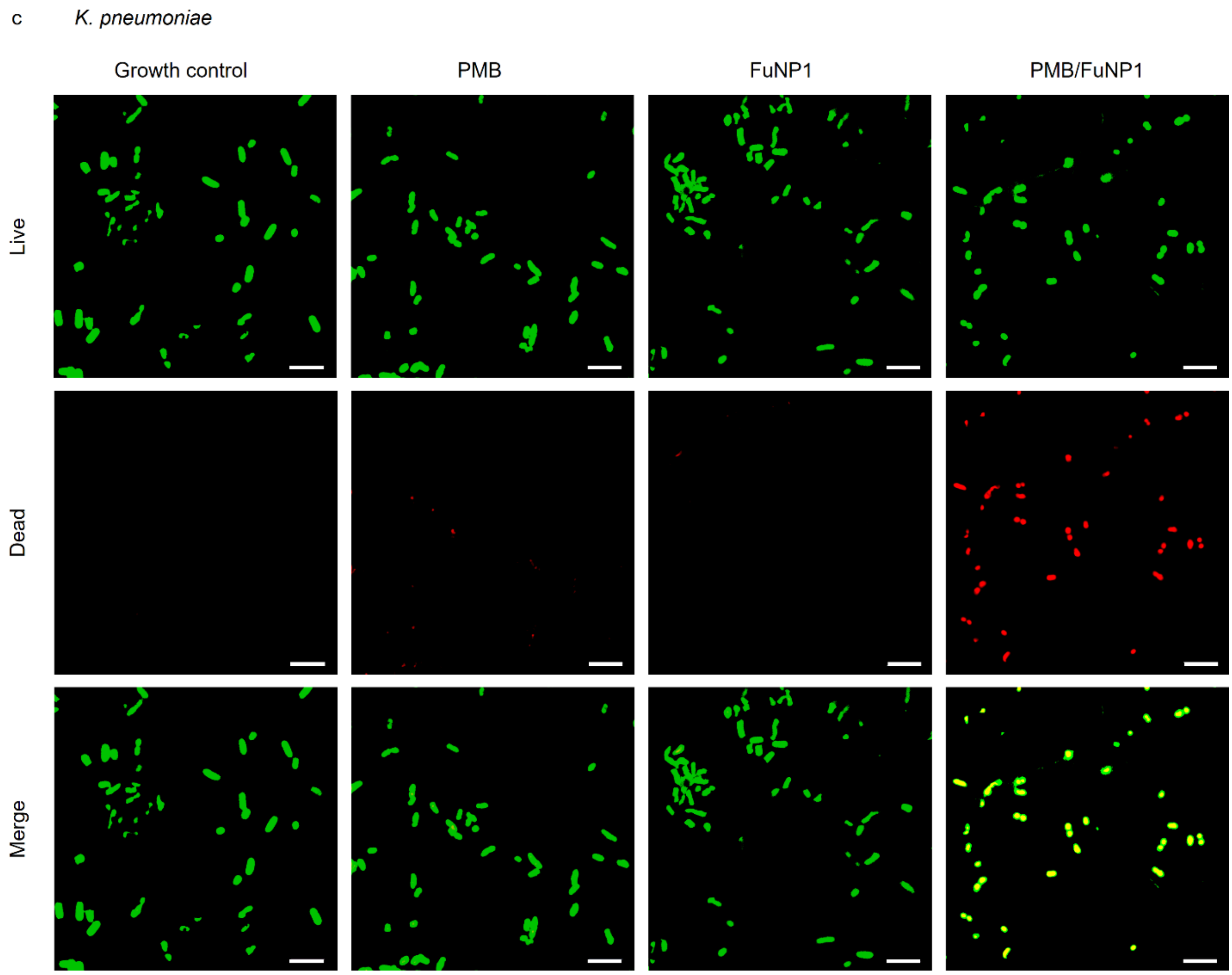
c
K. pneumoniae
Growth control
PMB
FuNP1
PMB/FuNP1
Live
Dead
Merge

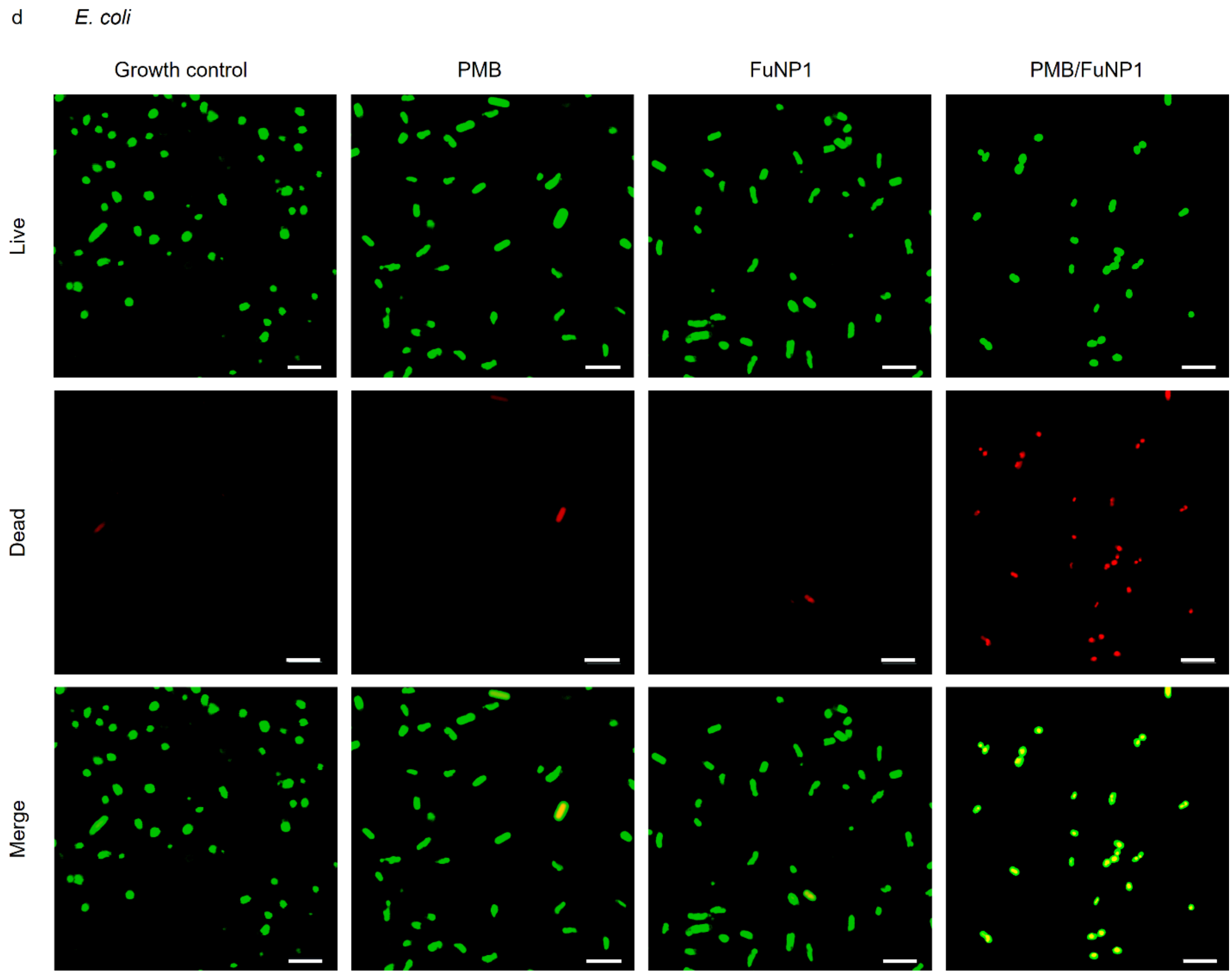
d
E. coli
Growth control
PMB
FuNP1
PMB/FuNP1
Live
Dead
Merge

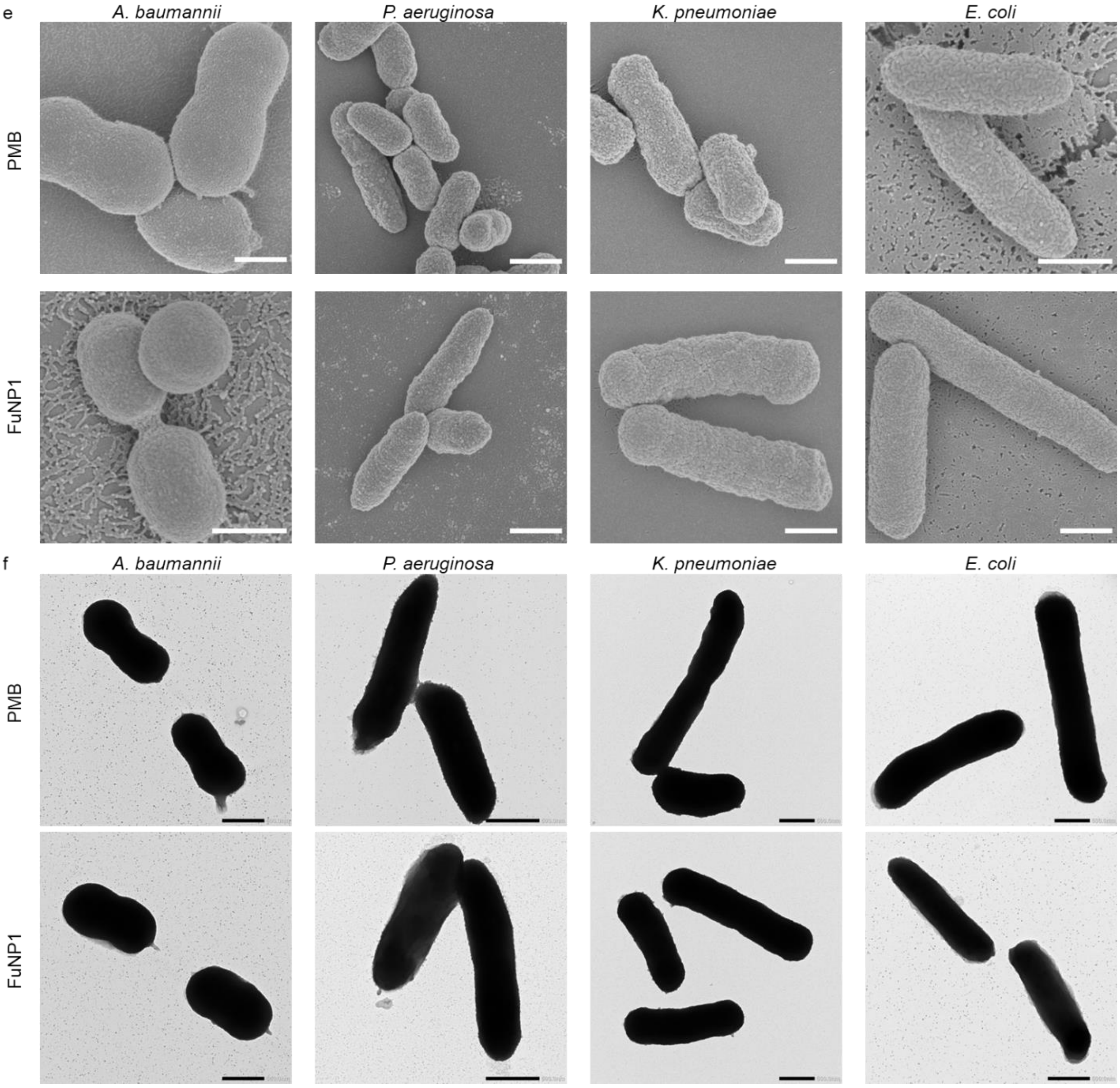

Figure S7. Visualization of the changes of bacteria after incubation with polymyxin B/FuNP1 *in vitro*. a-d) Representative CLSM fluorescence images for a live/dead assay of 4 Gram-negative bacteria after incubation with polymyxin B/FuNP1 at 37 °C for 4h. Scale bar: 5 μm. e) SEM images of 4 Gram-negative bacteria after polymyxin B/FuNP1 treatment at 37 °C for 4h. Scale bar: 500 nm. f) TEM images of 4 Gram-negative bacteria after polymyxin B/FuNP1 treatment at 37 °C for 4h. Scale bar: 500 nm.

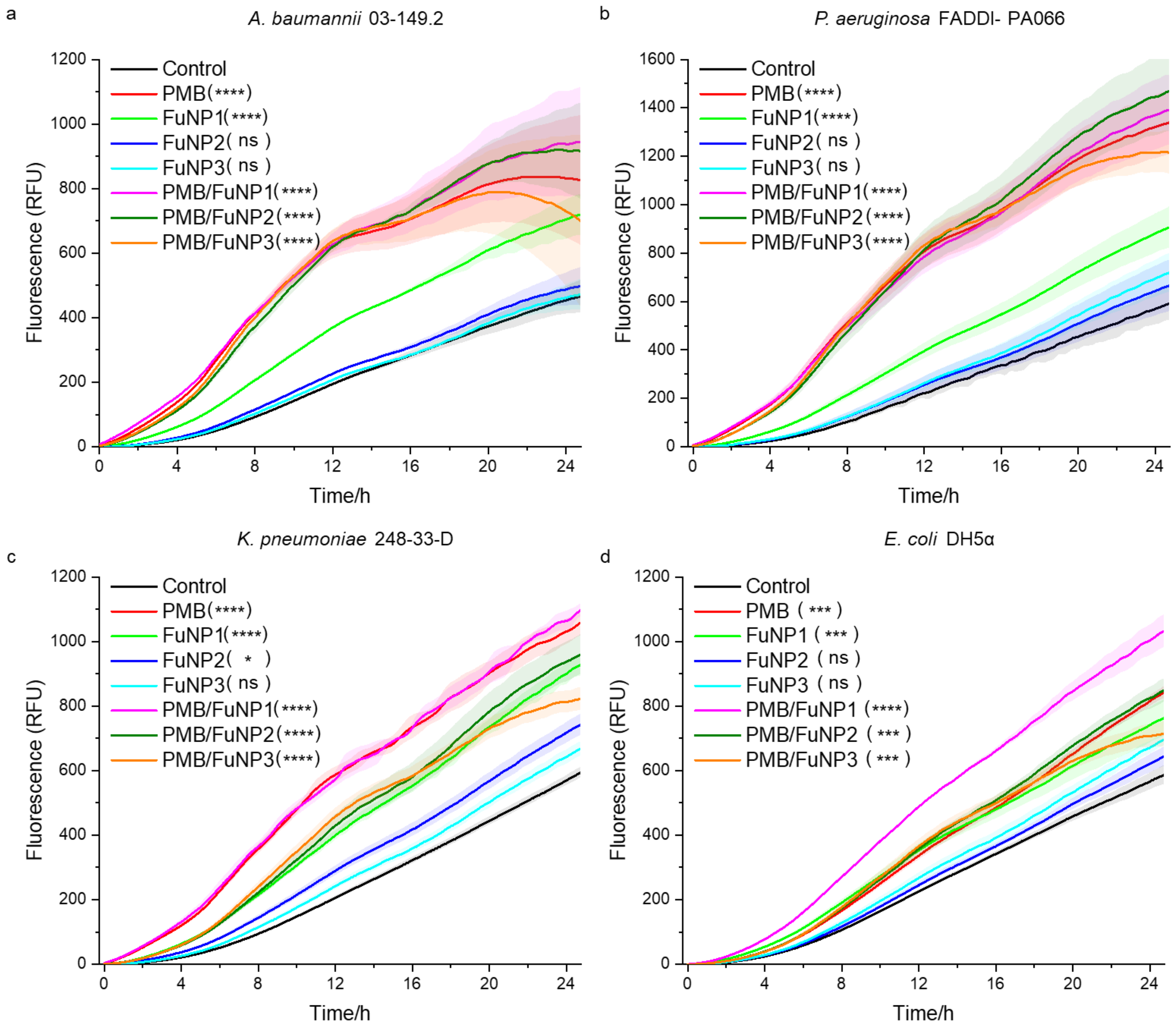

Figure S8. PMB/FuNPs increased membrane permeability. The relative fluorescence units (RFU) (n = 3) generated over time under different treatments in 2',7'-dichlorofluorescin diacetate-stained a) *A. baumannii* 03-149.2, b) *P. aeruginosa* FADDI- PA066, c) *K. pneumoniae* 248-33-D and d) *E. coli* DH5α. The concentrations used for PMB was 1/4×MIC, and 128 μg/mL for FuNPs. Statistical significance was determined with a Student's t-test. *$p < 0.05$, ***$p < 0.001$, ****$p < 0.0001$.

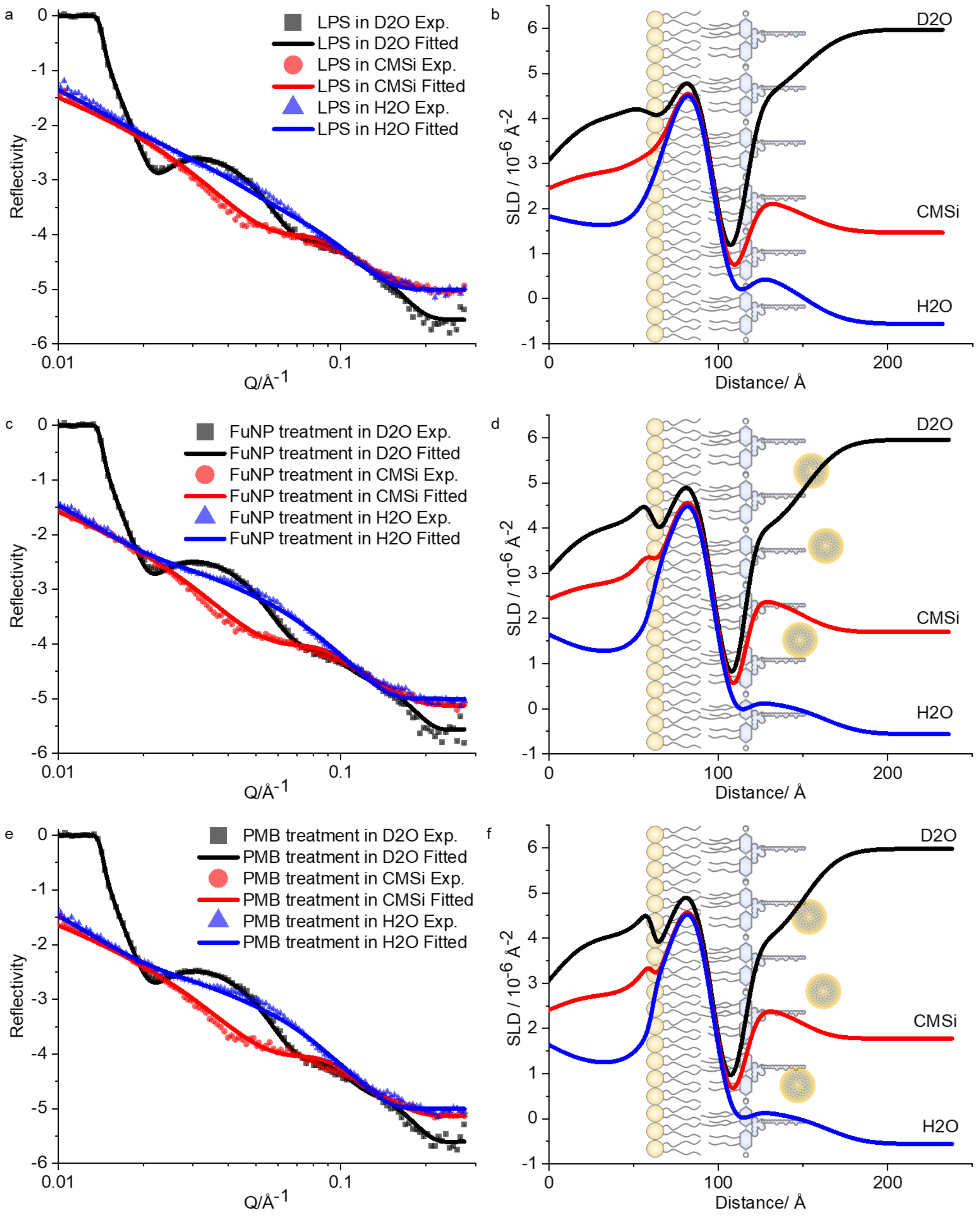

Figure S9. Neutron reflectometry profiles for LPS bilayer treated with FuNP followed by PMB. Experimental (Exp., symbols) and fitted (solid lines) profiles in D_2O (black squares), CMSi (red circles) and H_2O (blue up triangle). **a**) LPS bilayer; **b**) the corresponding scattering length density (SLD) profiles of (**a**); **c**) LPS bilayer treated with 128 µg/mL FuNP; **d**) the corresponding SLD profiles of (**c**); **e**) Followed by treatment with 4 µg/mL PMB; **f**) the corresponding SLD profiles of (**e**). For each condition in neutron reflectometry, we conducted multiple isotopic contrasts (D_2O, CMSi and H_2O) which would remove any artifacts in the fitting. Error bars for experimental data points is the standard deviation of the number of counts.

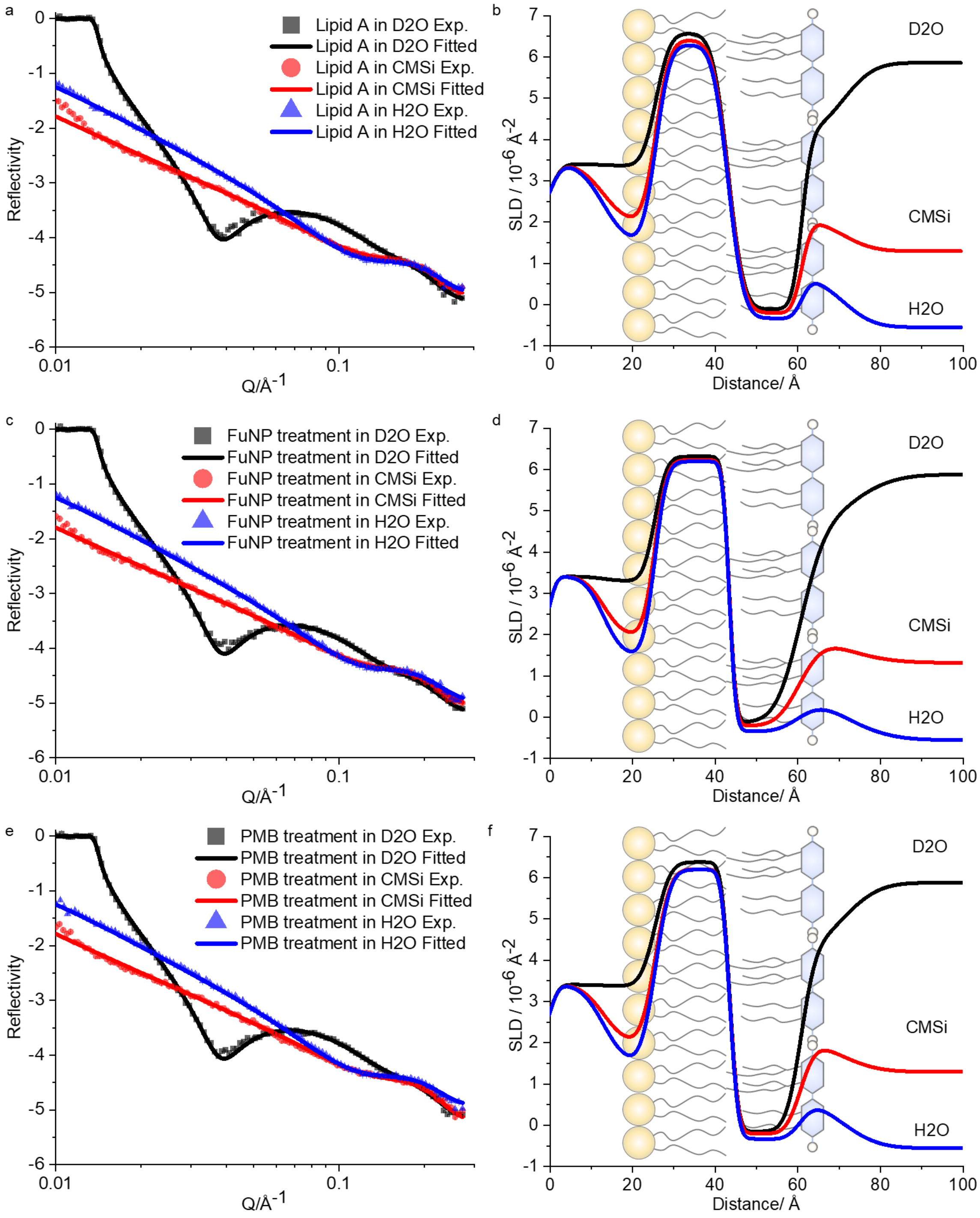

Figure S10. Neutron reflectometry profiles for lipid A bilayer treated with FuNP followed by PMB. Experimental (Exp., symbols) and fitted (solid lines) profiles in D_2O (black squares), CMSi (red circles) and H_2O (blue up triangle). **a**) Lipid A bilayer; **b**) the corresponding scattering length density (SLD) profiles of (**a**); **c**) Lipid A bilayer treated with 128 μg/mL FuNP; **d**) the corresponding SLD profiles of (**c**); **e**) Followed by treatment with 4 μg/mL PMB; **f**) the corresponding SLD profiles of (**e**). For each condition in neutron reflectometry, we conducted multiple isotopic contrasts (D_2O, CMSi and H_2O) which would remove any artifacts in the fitting. Error bars for experimental data points is the standard deviation of the number of counts.

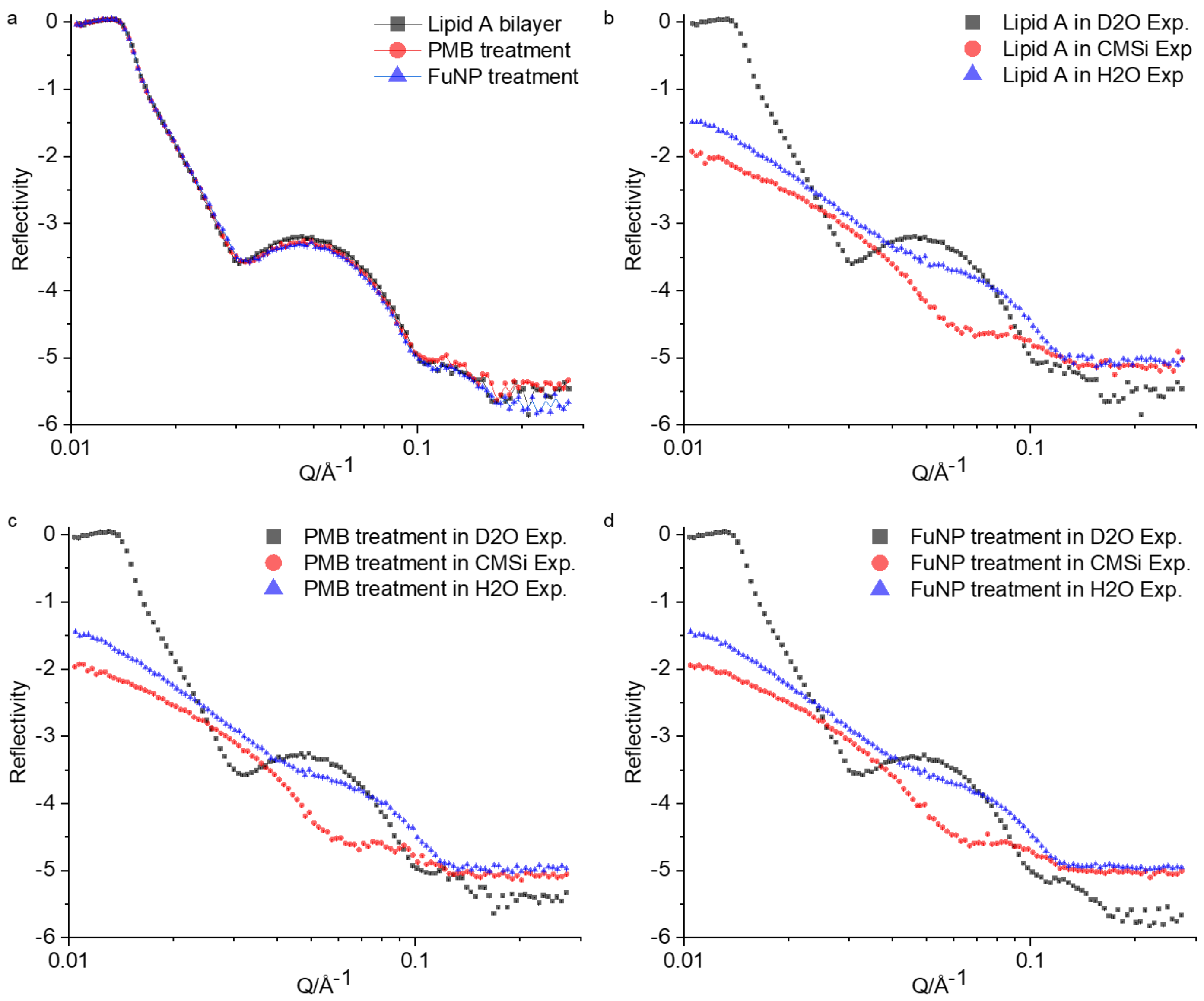

Figure S11. Neutron reflectometry profiles for lipid A bilayer treated with PMB followed by FuNP. Experimental (Exp., symbols) profiles in D_2O (black squares), CMSi (red circles) and H_2O (blue up triangle). **a**) The comparison of lipid A bilayer (black square) treated with PMB (red circle), followed by FuNP treatment (blue up triangle). **b**) Lipid A bilayer; **c**) Lipid A bilayer treated with PMB; **d**) Followed by treatment with FuNP. For each condition in neutron reflectometry, we conducted multiple isotopic contrasts (D_2O, CMSi and H_2O) which would remove any artifacts in the fitting. Error bars for experimental data points is the standard deviation of the number of counts.

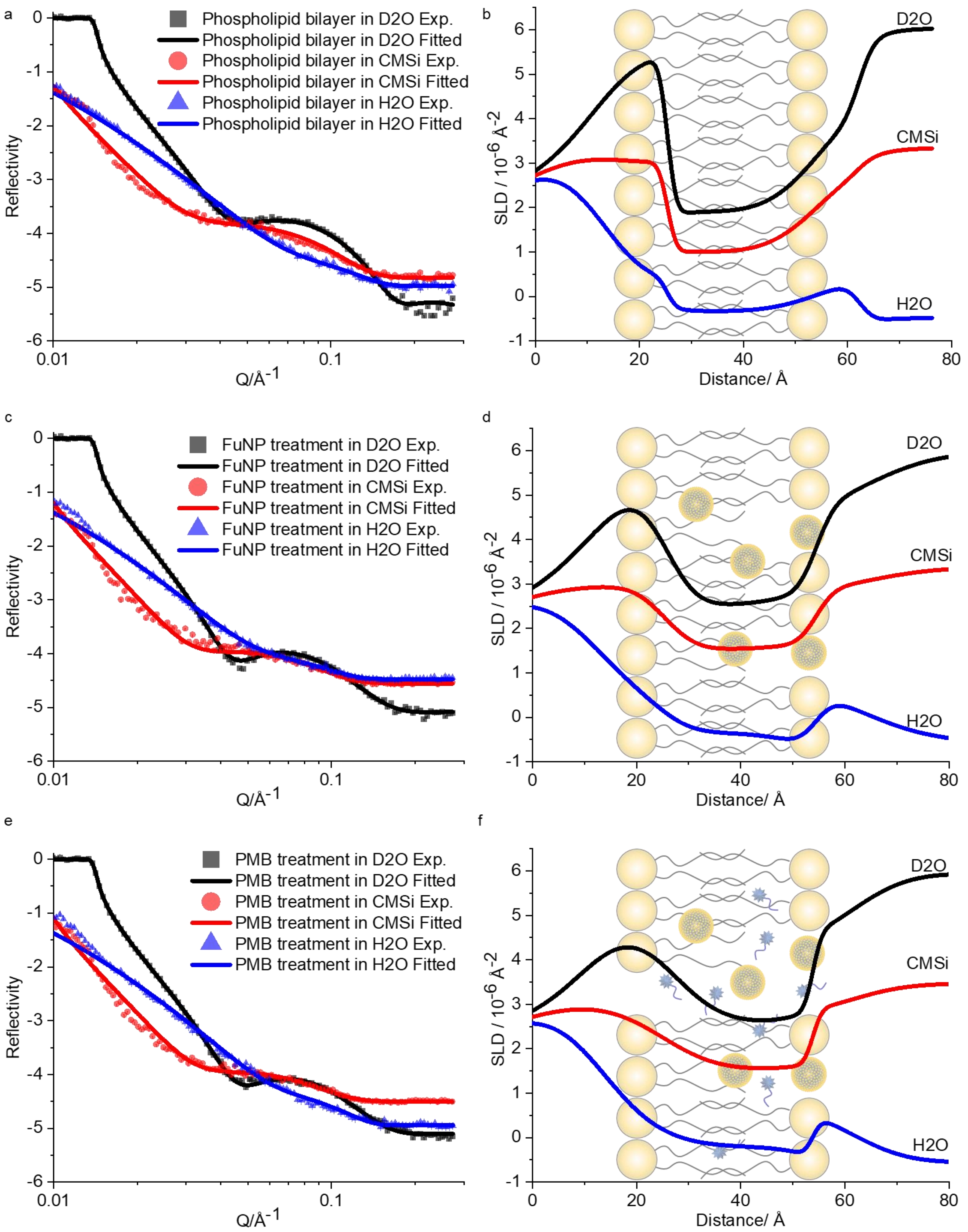

Figure S12. Neutron reflectometry profiles for phospholipid bilayer treated with FuNP followed by PMB. Experimental (Exp., symbols) and fitted (solid lines) profiles in D_2O (black squares), CMSi (red circles) and H_2O (blue up triangle). **a**) Phospholipid bilayer; **b**) the corresponding scattering length density (SLD) profiles of (**a**); **c**) Phospholipid bilayer treated with 128 μg/mL FuNP; **d**) the corresponding SLD profiles of (**c**); **e**) Followed by treatment with 4 μg/mL PMB; **f**) the corresponding SLD profiles of (**e**). For each condition in neutron reflectometry, we conducted multiple isotopic contrasts (D_2O, CMSi and H_2O) which would remove any artifacts in the fitting. Error bars for experimental data points is the standard deviation of the number of counts.

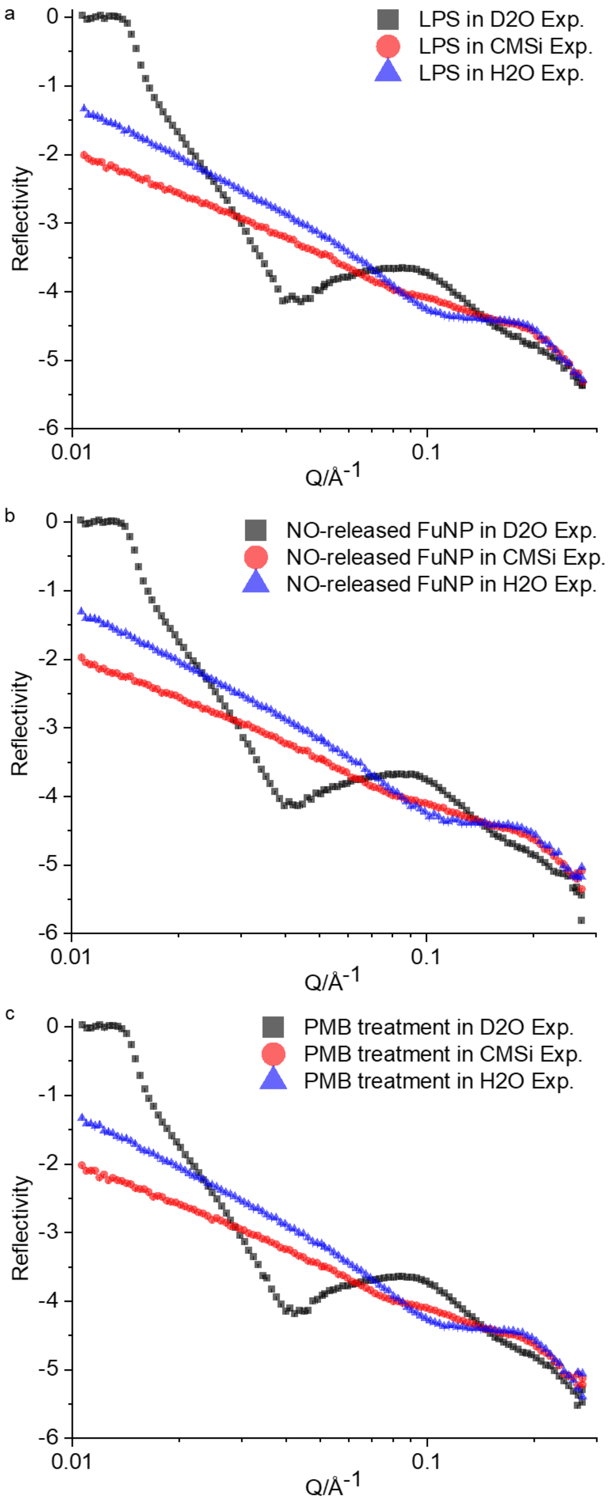

Figure S13. Neutron reflectometry profiles for LPS bilayer treated with NO-released FuNP followed by PMB. Experimental (Exp., symbols) and fitted (solid lines) profiles in D_2O (black squares), CMSi (red circles) and H_2O (blue up triangle). **a**) LPS bilayer; **b**) LPS bilayer treated with 128 μg/mL NO-released FuNP; **c**) Followed by treatment with 4 μg/mL PMB. For each condition in neutron reflectometry, we conducted multiple isotopic contrasts (D_2O, CMSi and H_2O) which would remove any artifacts in the fitting. Error bars for experimental data points is the standard deviation of the number of counts.

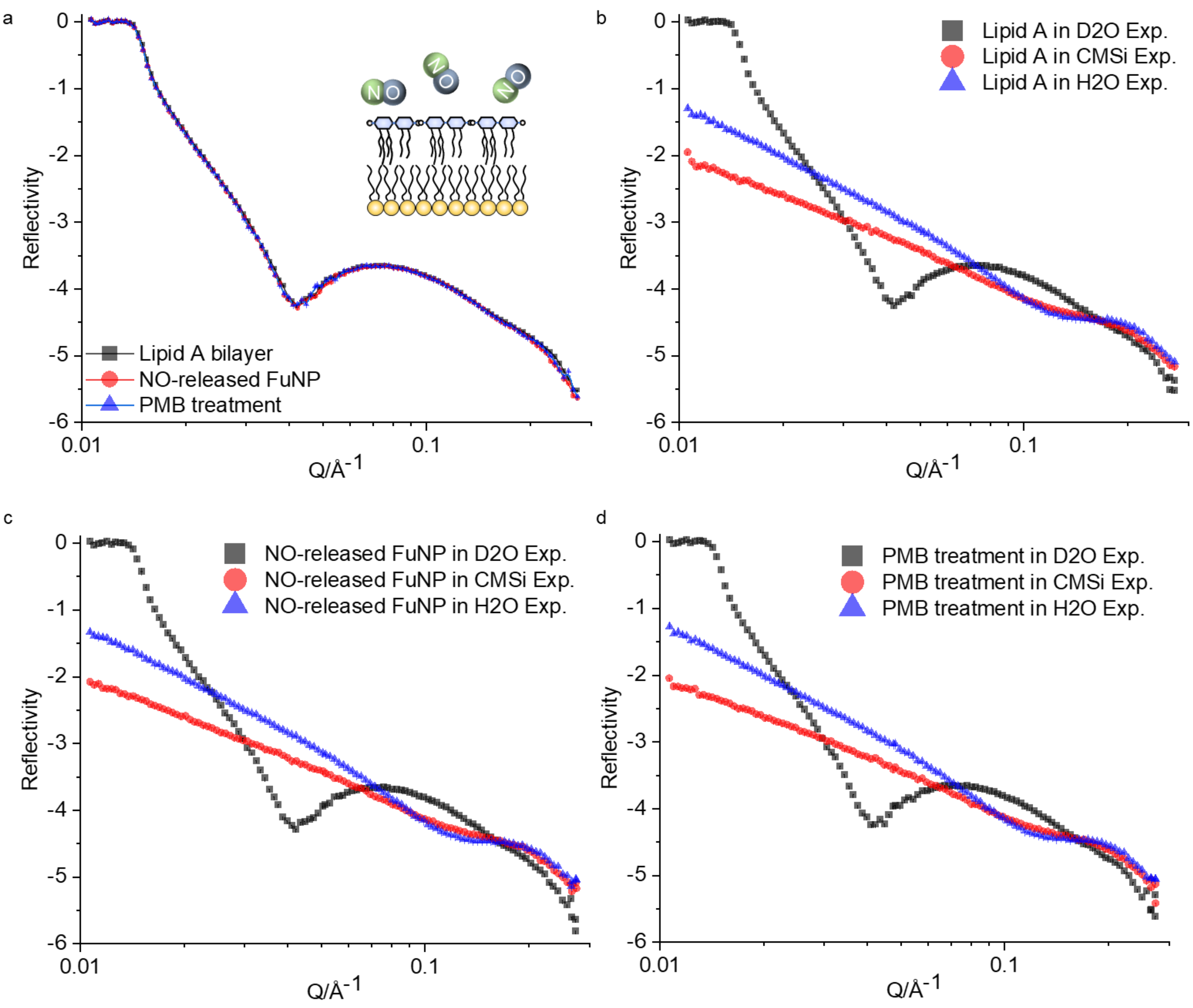

Figure S14. Neutron reflectometry profiles for lipid A bilayer treated with NO-released FuNP followed by PMB. Experimental (Exp., symbols) profiles in D_2O (black squares), CMSi (red circles) and H_2O (blue up triangle). **a**) The comparison of wildtype lipid A bilayer (black curve) treated with NO-released FuNP (red curve), followed by PMB treatment (blue curve). **b**) Wildtype lipid A bilayer; **c**) Lipid A bilayer treated with 128 µg/mL NO-released FuNP; **d**) Followed by treatment with 4 µg/mL PMB. For each condition in neutron reflectometry, we conducted multiple isotopic contrasts (D_2O, CMSi and H_2O) which would remove any artifacts in the fitting. Error bars for experimental data points is the standard deviation of the number of counts.

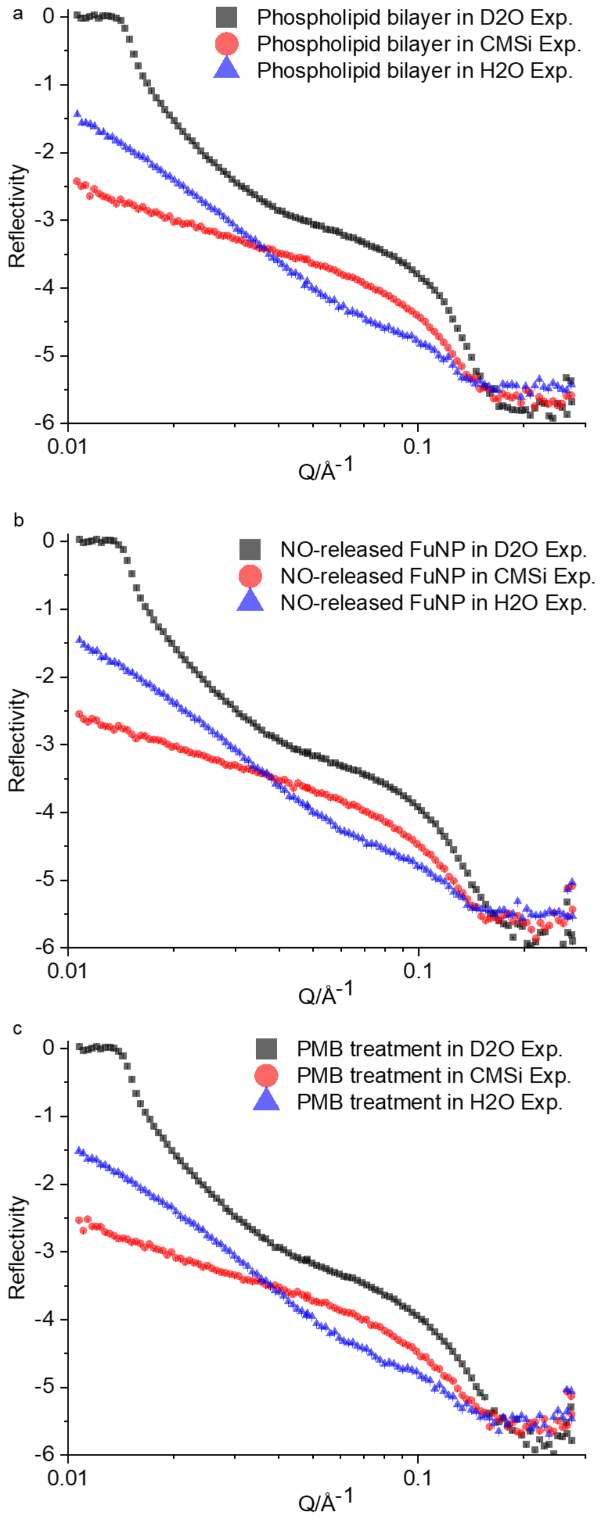

Figure S15. Neutron reflectometry profiles for phospholipid bilayer treated with NO-released FuNP followed by PMB. Experimental (Exp., symbols) and fitted (solid lines) profiles in D_2O (black squares), CMSi (red circles) and H_2O (blue up triangle). **a**) Phospholipid bilayer; **b**) Phospholipid bilayer treated with 128 µg/mL NO-released FuNP; **c**) Followed by treatment with 4 µg/mL PMB. For each condition in neutron reflectometry, we conducted multiple isotopic contrasts (D_2O, CMSi and H_2O) which would remove any artifacts in the fitting. Error bars for experimental data points is the standard deviation of the number of counts.

Table S1. Stability test of FuNP1 across 72 h.

Time (h)	Size (d, nm)	polydispersity	Zeta potential(mv)
0	427.6 ± 28.0	0.54 ± 0.08	-13.9 ± 1.5
1	411.9 ± 36.8	0.45 ± 0.04	-14.6 ± 1.2
4	416.9 ± 37.0	0.50 ± 0.04	-13.8 ± 0.3
8	438.5 ± 10.5	0.53 ± 0.11	-16.2 ± 1.5
12	413.0 ± 49.4	0.48 ± 0.11	-14.6 ± 0.9
24	424.5 ± 28.1	0.48 ± 0.08	-15.1 ± 1.8
48	418.4 ±2 4.8	0.47 ± 0.14	-14.7 ± 1.2
72	447.5 ± 8.1	0.53 ± 0.14	-15.1 ± 1.3

Table S2. Stability test of FuNP2 across 72 h.

Time (h)	Size (d, nm)	polydispersity	Zeta potential(mv)
0	483.5 ± 12.1	0.31 ± 0.03	-5.9 ± 0.4
1	480.7 ± 5.1	0.29 ± 0.05	-5.7 ± 0.4
4	473.4 ± 20.5	0.33 ± 0.01	-5.2 ± 0.3
8	484.8 ± 9.4	0.40 ± 0.09	-5.9 ± 0.4
12	465.9± 11.1	0.33 ± 0.05	-5.6 ± 0.1
24	470.6 ± 14.7	0.37 ± 0.09	-5.7 ± 0.4
48	468.2 ± 16.0	0.30 ± 0.04	-5.8 ± 0.4
72	462.1 ± 12.1	0.29 ± 0.07	-4.9 ± 0.3

Table S3. Stability test of FuNP3 across 72 h.

Time (h)	Size (d, nm)	polydispersity	Zeta potential(mv)
0	533.6 ± 10.5	0.36 ± 0.12	-3.6 ± 0.8
1	546.0 ± 20.4	0.43 ± 0.05	-2.7 ± 0.4
4	533.1 ± 11.2	0.47 ± 0.04	-4.1 ± 0.1
8	549.8 ± 32.1	0.46 ± 0.03	-3.3 ± 0.4
12	547.5 ± 26.4	0.43 ± 0.03	-3.1 ± 0.2
24	523.4 ± 6.1	0.44 ± 0.04	-3.4 ± 0.2
48	519.7 ± 8.9	0.56 ± 0.10	-3.0 ± 0.4
72	527.6 ± 2.8	0.45 ± 0.02	-3.7 ± 0.7

Table S4. Bacterial strains used in this study and the minimum inhibitory concentration (MIC) of polymyxin B (PMB) and FuNPs against these strains.

	Bacterial strains	MIC (µg/mL)				
		PMB	¼ PMB	FuNP-1	FuNP-2	FuNP-3
A. baumannii	03-149.2	256	64	512	512	512
	172	128	32	512	512	512
	03-149.1	1	0.25	512	512	> 512
K. pneumoniae	FADDI-KP003	512	128	> 512	512	> 512
	FADDI-KP027	256	64	512	512	> 512
	248-33-D	32	8	> 512	> 512	> 512
	B2	16	4	> 512	> 512	> 512
	B5055 KPN_01295	8	2	256	256	> 512
	RH201207	8	2	> 512	512	> 512
	II-503	8	2	> 512	> 512	> 512
	B5055	4	1	512	512	> 512
	NTUH-K2044	4	1	> 512	> 512	> 512
	NTUH-K2044 Δrfah	4	1	> 512	> 512	> 512
	B5055nm	2	0.5	512	512	> 512
	B5055 Δwza-cΔwaaF	0.25	0.0625	256	128	256
P. aeruginosa	FADDI- PA066	256	64	> 512	> 512	> 512
	FADDI-PA070	32	8	> 512	> 512	> 512
	FADDI-PA067	8	2	> 512	> 512	> 512
E. coli	DC10B	0.5	0.125	256	256	512
	DH5α	0.5	0.125	256	256	512
	JM109	0.5	0.125	512	512	> 512
	Top10	0.5	0.125	256	256	512
	G102	0.5	0.125	256	256	512

Table S5. Antibacterial activity of polymyxin B (PMB)/FuNP-1 against Gram-negative bacteria.

	Bacterial strains	MIC[a] (ug/mL)		FIC[b] (ug/mL)		FICI[d]	Outcome[e]
		PMB	FuNP1[c]	PMB	FuNP1		
A. baumannii	03-149.2	256	512	64	128	0.500	synergy
	172	128	512	32	128	0.500	synergy
	03-149.1	1	512	0.25	64	0.375	synergy
K. pneumoniae	FADDI-KP003	512	> 512	128	128	0.375	synergy
	FADDI-KP027	256	512	64	128	0.500	synergy
	248-33-D	32	> 512	8	16	0.266	synergy
	B2	16	> 512	4	32	0.281	synergy
	B5055 KPN_01295	8	256	2	32	0.375	synergy
	RH201207	8	> 512	2	32	0.281	synergy
	II-503	8	> 512	2	32	0.281	synergy
	B5055	4	512	1	16	0.281	synergy
	NTUH-K2044	4	> 512	1	128	0.375	synergy
	NTUH-K2044 Δrfah	4	> 512	1	128	0.375	synergy
	B5055nm	2	512	0.5	32	0.313	synergy
	B5055 Δwza-cΔwaaF	0.25	256	0.0625	64	0.500	synergy
P. aeruginosa	FADDI- PA066	256	> 512	64	4	0.254	synergy
	FADDI-PA070	32	> 512	8	32	0.281	synergy
	FADDI-PA067	8	> 512	2	4	0.254	synergy
E. coli	DC10B	0.5	256	0.125	64	0.500	synergy
	DH5α	0.5	256	0.125	64	0.500	synergy
	JM109	0.5	512	0.125	128	0.500	synergy
	Top10	0.5	256	0.125	64	0.500	synergy
	G102	0.5	256	0.125	64	0.500	synergy

[a]Minimum inhibitory concentration (MIC); [b]Fractional inhibitory concentration (FIC); [c]The endpoint MIC value could not be determined due to MIC > 512 μg/mL, the next MIC value (1024 ug/mL) was used for the calculation; [d]FIC index (FICI) = (FIC_{A1}/MIC_{A1}) + (FIC_{A2}/MIC_{A2}), where A1 = polymyxin B and A2 = FuNP-1; [e]Synergy was defined as FICI≤ 0.5.

Table S6. Antibacterial activity of polymyxin B (PMB)/FuNP-2 against Gram-negative bacteria.

	Bacterial strains	MIC[a] (ug/mL)		FIC[b] (ug/mL)		FICI[d]	Outcome[e]
		PMB	FuNP2[c]	PMB	FuNP2		
A. baumannii	03-149.2	256	512	64	128	0.500	synergy
	172	128	512	32	128	0.500	synergy
	03-149.1	1	512	0.25	32	0.313	synergy
K. pneumoniae	FADDI-KP003	512	512	128	64	0.375	synergy
	FADDI-KP027	256	512	64	64	0.375	synergy
	248-33-D	32	> 512	8	16	0.266	synergy
	B2	16	> 512	4	16	0.266	synergy
	B5055 KPN_01295	8	256	2	32	0.375	synergy
	RH201207	8	512	2	32	0.313	synergy
	II-503	8	> 512	2	16	0.266	synergy
	B5055	4	512	1	8	0.266	synergy
	NTUH-K2044	4	> 512	1	128	0.375	synergy
	NTUH-K2044 Δrfah	4	> 512	1	128	0.375	synergy
	B5055nm	2	512	0.5	8	0.266	synergy
	B5055 Δwza-cΔwaaF	0.25	128	0.0625	32	0.500	synergy
P. aeruginosa	FADDI- PA066	256	> 512	64	4	0.254	synergy
	FADDI-PA070	32	> 512	8	32	0.281	synergy
	FADDI-PA067	8	> 512	2	4	0.254	synergy
E. coli	DC10B	0.5	256	0.125	64	0.500	synergy
	DH5α	0.5	256	0.125	32	0.375	synergy
	JM109	0.5	512	0.125	128	0.500	synergy
	Top10	0.5	256	0.125	64	0.500	synergy
	G102	0.5	256	0.125	32	0.375	synergy

[a]Minimum inhibitory concentration (MIC); [b]Fractional inhibitory concentration (FIC); [c]The endpoint MIC value could not be determined due to MIC > 512 μg/mL, the next MIC value (1024 ug/mL) was used for the calculation; [d]FIC index (FICI) = (FIC_{A1}/MIC_{A1}) + (FIC_{A2}/MIC_{A2}), where A1 = PMB and A2 = FuNP-2; [e]Synergy was defined as FICI≤ 0.5.

Table S7. Antibacterial activity of polymyxin B (PMB)/FuNP-3 against Gram-negative bacteria.

	Bacterial strains	MIC[a] (ug/mL)		FIC[b] (ug/mL)		FICI[d]	Outcome[e]
		PMB	FuNP3[c]	PMB	FuNP3		
A. baumannii	03-149.2	256	512	64	64	0.375	synergy
	172	128	512	32	64	0.375	synergy
	03-149.1	1	> 512	0.25	128	0.375	synergy
K. pneumoniae	FADDI-KP003	512	> 512	128	32	0.281	synergy
	FADDI-KP027	256	> 512	64	128	0.375	synergy
	248-33-D	32	> 512	8	4	0.254	synergy
	B2	16	> 512	4	8	0.258	synergy
	B5055 KPN_01295	8	> 512	2	16	0.266	synergy
	RH201207	8	> 512	2	32	0.281	synergy
	II-503	8	> 512	2	32	0.281	synergy
	B5055	4	> 512	1	8	0.258	synergy
	NTUH-K2044	4	> 512	1	128	0.375	synergy
	NTUH-K2044 Δrfah	4	> 512	1	128	0.375	synergy
	B5055nm	2	> 512	0.5	64	0.313	synergy
	B5055 Δwza-cΔwaaF	0.25	256	0.0625	64	0.500	synergy
P. aeruginosa	FADDI- PA066	256	> 512	64	16	0.266	synergy
	FADDI-PA070	32	> 512	8	4	0.254	synergy
	FADDI-PA067	8	> 512	2	8	0.258	synergy
E. coli	DC10B	0.5	512	0.125	32	0.313	synergy
	DH5α	0.5	512	0.125	64	0.375	synergy
	JM109	0.5	> 512	0.125	128	0.375	synergy
	Top10	0.5	512	0.125	64	0.375	synergy
	G102	0.5	512	0.125	64	0.375	synergy

[a]Minimum inhibitory concentration (MIC); [b]Fractional inhibitory concentration (FIC); [c]The endpoint MIC value could not be determined due to MIC > 512 µg/mL, the next MIC value (1024 ug/mL) was used for the calculation; [d]FIC index (FICI) = (FIC_{A1}/MIC_{A1}) + (FIC_{A2}/MIC_{A2}), where A1 = PMB and A2 = FuNP-3; [e]Synergy was defined as FICI≤ 0.5.

Table S8. Antibacterial activity of polymyxin B (PMB)/ bulk-form furoxan against Gram-negative bacteria.

Bacterial strains	Fractional inhibitory concentration (ug/mL)			
	PMB	Furoxan 1	Furoxan 2	Furoxan 3
A. baumannii 03-149.2	64	256	256	128
A. baumannii 172	32	256	256	256
K. pneumoniae 248-33-D	8	64	32	32
K. pneumoniae B2	4	32	32	16
P. aeruginosa FADDI- PA066	64	16	16	32
P. aeruginosa FADDI-PA067	2	16	16	16
E. coli DC10B	0.125	64	128	64
*E. coli*DH5α	0.125	64	128	128

Table S9. Antibacterial activity of polymyxin B (PMB)/Ampicillin against Gram-negative bacteria.

Bacterial strains	MIC[a] (ug/mL)		FIC[b] (ug/mL)		FICI[d]	Outcome[e]
	PMB	Ampicillin [c]	PMB	Ampicillin		
A. baumannii 03-149.2	256	> 256	64	2	0.254	synergy
A. baumannii 03-149.1	1	> 256	0.25	> 256	1.250	indifference
K. pneumoniae 248-33-D	32	4	8	2	0.750	indifference
K. pneumoniae B5055	4	1	1	4	4.250	indifference
P. aeruginosa FADDI- PA066	256	128	64	4	0.281	synergy
P. aeruginosa FADDI-PA067	8	128	2	64	0.750	indifference
E. coli DC10B	0.5	2	0.125	1	0.750	indifference
E. coli DH5α	0.5	> 256	0.125	> 256	1.250	indifference

[a]Minimum inhibitory concentration (MIC); [b]Fractional inhibitory concentration (FIC); [c]The endpoint MIC value could not be determined due to MIC > 256 μg/mL, the next MIC value (512 ug/mL) was used for the calculation; [d]FIC index (FICI) = (FIC_{A1}/MIC_{A1}) + (FIC_{A2}/MIC_{A2}), where A1 = PMB and A2 = Ampicillin; [e]synergy: FICI≤ 0.5, indifference: 0.5 < FICI < 4.

Table S10. Antibacterial activity of polymyxin B (PMB)/Aztreonam against Gram-negative bacteria.

Bacterial strains	MIC[a] (ug/mL)		FIC[b] (ug/mL)		FICI[d]	Outcome[e]
	PMB	Aztreonam [c]	PMB	Aztreonam		
A. baumannii 03-149.2	256	64	64	0.064	0.251	synergy
A. baumannii 03-149.1	1	128	0.25	> 256	4.250	indifference
K. pneumoniae 248-33-D	32	128	8	4	0.281	synergy
K. pneumoniae B5055	4	0.25	1	0.064	0.506	indifference
P. aeruginosa FADDI- PA066	256	8	64	0.125	0.266	synergy
P. aeruginosa FADDI-PA067	8	> 256	2	16	0.281	synergy
E. coli DC10B	0.5	0.25	0.125	0.125	0.750	indifference
E. coli DH5α	0.5	32	0.125	32	1.250	indifference

[a]Minimum inhibitory concentration (MIC); [b]Fractional inhibitory concentration (FIC); [c]The endpoint MIC value could not be determined due to MIC > 256 μg/mL, the next MIC value (512 ug/mL) was used for the calculation; [d]FIC index (FICI) = (FIC_{A1}/MIC_{A1}) + (FIC_{A2}/MIC_{A2}), where A1 = PMB and A2 = Ampicillin; [e]synergy: FICI≤ 0.5, indifference: 0.5 < FICI < 4.

Table S11. Antibacterial activity of polymyxin B (PMB)/ Doripenem against Gram-negative bacteria.

Bacterial strains	MIC[a] (ug/mL)		FIC[b] (ug/mL)		FICI[d]	Outcome[e]
	PMB	Doripenem [c]	PMB	Doripenem		
A. baumannii 03-149.2	256	32	64	0.125	0.254	synergy
A. baumannii 03-149.1	1	32	0.25	32	1.250	indifference
K. pneumoniae 248-33-D	32	0.125	8	0.064	0.762	indifference
K. pneumoniae B5055	4	0.125	1	0.064	0.762	indifference
P. aeruginosa FADDI- PA066	256	8	64	0.125	0.266	synergy
P. aeruginosa FADDI-PA067	8	32	2	0.5	0.266	synergy
E. coli DC10B	0.5	0.125	0.125	0.064	0.762	indifference
E. coli DH5α	0.5	16	0.125	16	1.250	indifference

[a]Minimum inhibitory concentration (MIC); [b]Fractional inhibitory concentration (FIC); [c]The endpoint MIC value could not be determined due to MIC > 256 μg/mL, the next MIC value (512 ug/mL) was used for the calculation; [d]FIC index (FICI) = (FIC_{A1}/MIC_{A1}) + (FIC_{A2}/MIC_{A2}), where A1 = PMB and A2 = Ampicillin; [e]synergy: FICI≤ 0.5, indifference: 0.5 < FICI < 4.

Table S12. Antibacterial activity of polymyxin B (PMB)/NO-released FuNPs against Gram-negative bacteria.

Bacterial strains	FIC[a] (µg/mL)		FICI[c]	Outcome[d]
	PMB	NO-released FuNPsI[b]		
A. baumannii 03-149.2	256	1~128	> 1	indifference
K. pneumoniae 248-33-D	32	1~128	> 1	indifference
P. aeruginosa FADDI- PA066	256	1~128	> 1	indifference
E. coli DH5α	0.5	1~128	> 1	indifference

[a]Fractional inhibitory concentration (FIC); [b]After NO releasing from FuNPs, PMB was the main factor to inhibit the bacterial growth with its corresponding minimum inhibitory concentration, while the FuNPs in the test range (1~ 128 µg/mL) did not make any significant difference to the combined inhibitory action; [c]FIC index (FICI) = (FIC_{A1}/MIC_{A1}) + (FIC_{A2}/MIC_{A2}), where A1 = PMB and A2 = FuNPs; [d]Indifference was defined as 0.5 < FICI < 4.

Table S13. Resistance acquisition (n = 2) of Gram-negative bacteria during serial passaging in the presence of MIC levels (µg/mL) of PMB in cation adjusted Mueller-Hinton broth.

Time/ day	*A. baumannii*	*P. aeruginosa*	*K. pneumoniae*	*E. coli*
0	256	256	32	0.5
1	256	256	32	1
2	512	512	32	4
3	512	1024	64	16
4	2048	1024	512	8
5	2048	1024	512	8
6	2048	2048	2048	8
7	2048	2048	>2048	16
8	>2048	>2048	>2048	16
9	>2048	>2048	>2048	16
10	>2048	>2048	>2048	16
11	>2048	>2048	>2048	16
12	>2048	>2048	>2048	16
13	>2048	>2048	>2048	32
14	>2048	>2048	>2048	32
15	>2048	>2048	>2048	32
16	>2048	>2048	>2048	32
17	>2048	>2048	>2048	32
18	>2048	>2048	>2048	32
19	>2048	>2048	>2048	32
20	>2048	>2048	>2048	32
21	>2048	>2048	>2048	32

Table S14. Resistance acquisition (n = 2) of Gram-negative bacteria during serial passaging in the presence of MIC levels of FuNPs in cation adjusted Mueller-Hinton broth.

Time	FuNP1, µg/mL				FuNP2, µg/mL				FuNP3, µg/mL			
/Day	AB[a]	PA[b]	KP[c]	EC[d]	AB[a]	PA[b]	KP[c]	EC[d]	AB[a]	PA[b]	KP[c]	EC[d]
0	512	>512	>512	256	512	>512	>512	256	512	>512	>512	512
1	512	>512	>512	512	512	>512	>512	256	512	>512	>512	512
2	512	>512	>512	512	512	>512	>512	512	>512	>512	>512	512
3	>512	>512	>512	512	512	>512	>512	512	>512	>512	>512	512
4	>512	>512	>512	512	512	>512	>512	512	>512	>512	>512	512
5	>512	>512	>512	512	>512	>512	>512	>512	>512	>512	>512	>512
6	>512	>512	>512	>512	>512	>512	>512	>512	>512	>512	>512	>512
7	>512	>512	>512	>512	>512	>512	>512	>512	>512	>512	>512	>512
8	>512	>512	>512	>512	>512	>512	>512	>512	>512	>512	>512	>512
9	>512	>512	>512	>512	>512	>512	>512	>512	>512	>512	>512	>512
10	>512	>512	>512	>512	>512	>512	>512	>512	>512	>512	>512	>512
11	>512	>512	>512	>512	>512	>512	>512	>512	>512	>512	>512	>512
12	>512	>512	>512	>512	>512	>512	>512	>512	>512	>512	>512	>512
13	>512	>512	>512	>512	>512	>512	>512	>512	>512	>512	>512	>512
14	>512	>512	>512	>512	>512	>512	>512	>512	>512	>512	>512	>512
15	>512	>512	>512	>512	>512	>512	>512	>512	>512	>512	>512	>512
16	>512	>512	>512	>512	>512	>512	>512	>512	>512	>512	>512	>512
17	>512	>512	>512	>512	>512	>512	>512	>512	>512	>512	>512	>512
18	>512	>512	>512	>512	>512	>512	>512	>512	>512	>512	>512	>512
19	>512	>512	>512	>512	>512	>512	>512	>512	>512	>512	>512	>512
20	>512	>512	>512	>512	>512	>512	>512	>512	>512	>512	>512	>512
21	>512	>512	>512	>512	>512	>512	>512	>512	>512	>512	>512	>512

[a]*A. baumannii*; [b]*P. aeruginosa*; [c]*K. pneumoniae*; [d]*E. coli*.

Table S15. Scattering length density (SLD) used to fit neutron reflectometry data.

Materials	SLD (× 10^{-6} Å^{-2})		
	D_2O[a]	CMSi[b]	H_2O[c]
Si	2.07	2.07	2.07
SiO_2	3.41	3.41	3.41
H_2O	-	-	-0.56
CMSi		2.07	
D_2O	6.35	-	-
DPPC head	1.98	1.98	1.98
d-DPPC tail	7.45	7.45	7.45
Lipid A tail	-0.39	-0.39	-0.39
Lipid A GlcN head	3.39	2.89	2.58
LPS head	4.28	2.87	2.01
LPS tail	-0.39	-0.39	-0.39
Polymyxin B	4.18	2.56	1.57
h-POPE head	2.11	2.11	2.11
h-POPE tail	-0.28	-0.28	-0.28
h-POPG head	3.59	3.09	2.78
h-POPG tail	-0.28	-0.28	-0.28
TOCL head	3.11	2.99	2.91
TOCL tail	-0.21	-0.21	-0.21
FuNP1	2.22	1.97	1.81

a)D_2O 100%; b)D_2O/H_2O (38/62, v/v), contrast matched the silicon substrate (CMSi); c)H_2O 100%. The SLD for monolaurin was calculated by: $\mathrm{SLD}=\frac{\rho N_a \sum_{i=1}^{N} b_i}{\sum_{i=1}^{N} M_i}$, where the scattering length contributions (b_i) from the N atoms within the unit cell are summed, ρ is the mass density of monolaurin, N_a is the Avogadro constant, and M_i is the atomic molar mass for each element. GlcN, glucosamine. DPPC, 1,2-dipalmitoyl(d_{62})-sn-glycero-3-phosphocholine.

Table S16. Fitted parameters for LPS: d-DPPC bilayer treated with FuNP1 followed by polymyxin B (PMB).

Sublayer	Thickness Å	$V_{d\text{-}DPPC}$[a] %	V_{LPS}[a] %	V_{FuNP1}[a] %	V_{PMB}[a] %	$V_{Hydration}$[a] %
d-DPPC head	14.7±0.6	97.9±1.4	-	-	-	2.1±1.4
d-DPPC tail	19.7±0.2					
LPS tail	19.5±0.5	-	95.9±2.7	-	-	4.1±2.7
LPS head	34.5±2.0					
FuNP1, 128 µg/mL						
d-DPPC head	14.7±0.1	97.9±1.4	-	-	-	2.1±1.4
d-DPPC tail	19.7±0.1					
LPS tail	19.6±0.1	-	95.9±2.7	-	-	4.1±2.7
LPS head	34.4±0.1					
FuNP1	5.6±0.3	-	-	15.6±1.6	-	84.4±1.6
PMB, 4 µg/mL						
d-DPPC head	14.7±0.2	97.9±1.4	-	-	-	2.1±1.4
d-DPPC tail	19.5±0.1					
LPS tail	19.6±0.2	-	95.9±1.4	-	-	4.1±1.4
LPS head	34.6±0.3					
FuNP1	5.3±0.4	-	-	15.5±1.5	-	84.5±1.5

[a]Volume fraction. All data are expressed as median ± s.d., based on values obtained from three isotopic contrasts fitted simultaneously using Monte Carlo analysis.

Table S17. Fitted parameters for Lipid A: d-DPPC bilayer treated with FuNP1 followed by polymyxin B (PMB).

Sublayer	Thickness Å	$V_{d\text{-}DPPC}$[a] %	$V_{Lipid\ A}$[a] %	V_{FuNP1}[a] %	V_{PMB}[a] %	$V_{Hydration}$[a] %
d-DPPC head	12.9±0.7	96.0±2.4		-	-	4.0±3.7
d-DPPC tail	17.4±0.2					
Lipid A tail	17.9±0.2	96.1±1.1		-	-	3.9±2.2
Lipid A head	9.9±0.6					
FuNP1, 128 µg/mL						
d-DPPC head	12.5±0.2	96.0±2.4		-	-	4.0±3.7
d-DPPC tail	17.9±01					
Lipid A tail	17.1±0.1	96.1±1.1		-	-	3.9±2.2
Lipid A head	9.5±0.3					
PMB, 4 µg/mL						
d-DPPC head	12.9±0.7	96.0±2.4		-	-	4.0±3.7
d-DPPC tail	17.4±0.2					
Lipid A tail	17.9±0.2	96.1±1.1		-	-	3.9±2.2
Lipid A head	9.9±0.6					

[a]Volume fraction. All data are expressed as median ± s.d., based on values obtained from three isotopic contrasts fitted simultaneously using Monte Carlo analysis.

Table S18. Fitted parameters for phospholipid bilayer treated with FuNP1 followed by polymyxin B (PMB).

Sublayer	Thickness/ Å	V_{lipid}[a]/ %	V_{FuNP1}[a]/ %	V_{PMB}[a] /%	$V_{Hydration}$[a]/ %
Inner head	12.9±0.1				
Tail	29.1±0.1	67.3±8.1	-	-	32.7±8.1
Outer head	7.8±0.1				
FuNP1, 128 μg/mL					
Inner head	13.4±0.1				
Tail	29.0±0.3	39.6±8.3	13.0±6.2	-	47.4±14.5
Outer head	7.9±0.3				
PMB, 4 μg/mL					
Inner head	12.5±0.1				
Tail	28.5±0.1	35.0±8.7	13.0±6.2	12.7±4.5	39.3±19.7
Outer head	7.5±0.1				

[a]Volume fraction. All data are expressed as median ± s.d., based on values obtained from three isotopic contrasts fitted simultaneously using Monte Carlo analysis.
